# Role of Side-Chain Free Volume on the Electrochemical Behavior of Poly(propylenedioxythiophenes)

**DOI:** 10.1021/acs.chemmater.3c02122

**Published:** 2024-03-12

**Authors:** Marlow M. Durbin, Alex H. Balzer, John R. Reynolds, Erin L. Ratcliff, Natalie Stingelin, Anna M. Österholm

**Affiliations:** † School of Chemical and Biomolecular Engineering, Georgia Institute of Technology, Atlanta, Georgia 30332, United States; ‡ School of Materials Science and Engineering, Georgia Institute of Technology, Atlanta, Georgia 30332, United States; § School of Chemistry and Biochemistry, Georgia Institute of Technology, Atlanta, Georgia 30332, United States; ∥ Department of Chemical and Environmental Engineering, The University of Arizona, Tucson, Arizona 85721-0012, United States

## Abstract

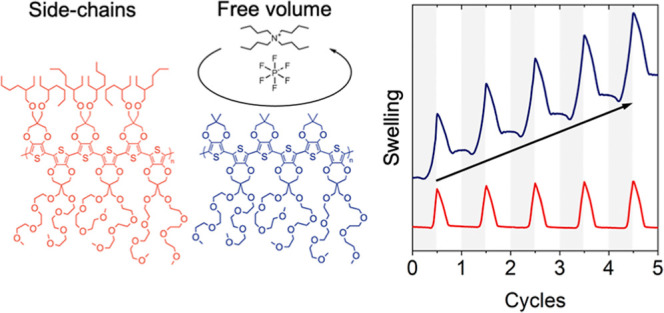

Mixed ionic/electronic conducting polymers are versatile systems for, e.g., energy storage, heat management (exploiting electrochromism), and biosensing, all of which require electrochemical doping, i.e., the electrochemical oxidation or reduction of their macromolecular backbones. Electrochemical doping is achieved via electro-injection of charges (i.e., electronic carriers), stabilized via migration of counterions from a supporting electrolyte. Since the choice of the polymer side-chain functionalization influences electrolyte and/or ion sorption and desorption, it in turn affects redox properties, and, thus, electrochemically induced mixed conduction. However, our understanding of how side-chain versus backbone design can increase ion flow while retaining high electronic transport remains limited. Hence, heuristic design approaches have typically been followed. Herein, we consider the redox and swelling behavior of three poly(propylenedioxythiophene) derivatives, P(ProDOT)s, substituted with different side-chain motifs, and demonstrate that passive swelling is controlled by the surface polarity of P(ProDOT) films. In contrast, active swelling under operando conditions (i.e., under an applied bias) is dictated by the local side-chain free volume on the length scale of a monomer unit. Such insights deliver important design criteria toward durable soft electrochemical systems for diverse energy and biosensing platforms and new understanding into electrochemical conditioning (“break-in”) in many conducting polymers.

Poly(propylenedioxythiophene)s, P(ProDOT)s, and their derivatives are a well-studied, chemically readily tunable class of conducting polymers for electrochemical applications with demonstrations in batteries,^1,2^ supercapacitors,^3,4^ electrochromic devices,^5,6^ organic electrochemical transistors,^7,8^ and biointerfaces. One promising P(ProDOT) derivative, poly(3,3-di(2,5,8,11-tetraoxadodecyl)-3′,3′-dimethyl-3,3′,4,4′-tetrahydro-2H,2′H-6,6′-bithieno[3,4-*b*][1,4]dioxepine), referred to here as P(OE3)-P(Me) (Figure 1a), is an alternating ProDOT copolymer that is asymmetrically substituted with two polar oligoether, OE3, side chains in the R_1_ positions, and two methyl, Me, moieties in the R_2_ positions. P(OE3)-P(Me) is electroactive in a variety of electrolytes, and displays a high gravimetric capacitance *C* > 80 F g^–1^ in, e.g., 0.1 M NaCl/H_2_O.^7^ Moreover, P(OE3)-P(Me) allows fabrication of organic electrochemical transistors, OECTs, that display On–Off ratios, *I*_On/Off_, in excess of 10^5^, a transconductance, *g*_m,max_, of 0.3 mS, and a μ C*, a critical OECT figure of merit given by the product of OECT device mobility, μ, and the device capacitance C*, of 57 ± 3 F cm^–1^ V^–1^ s^–1^.^8^ P(OE3)-P(Me) also exhibits reasonable electrochemical stability, allowing reversible and repeated cycling in 0.1 M NaCl/H_2_O over more than 1000 cycles.^8^ However, when using a lower dielectric constant electrolyte of tetrabutylammonium hexafluorophosphate, TBA^+^PF_6_^–^, dissolved in propylene carbonate, PC, a 35% loss of redox capacity over just 100 cycles was recorded (Figure 1b). In parallel, a notable decrease in the neutral-state optical density was found (Figure 1c)—not an uncommon phenomenon in many well-studied conducting polymers.^2,9,10^

**Figure 1:**
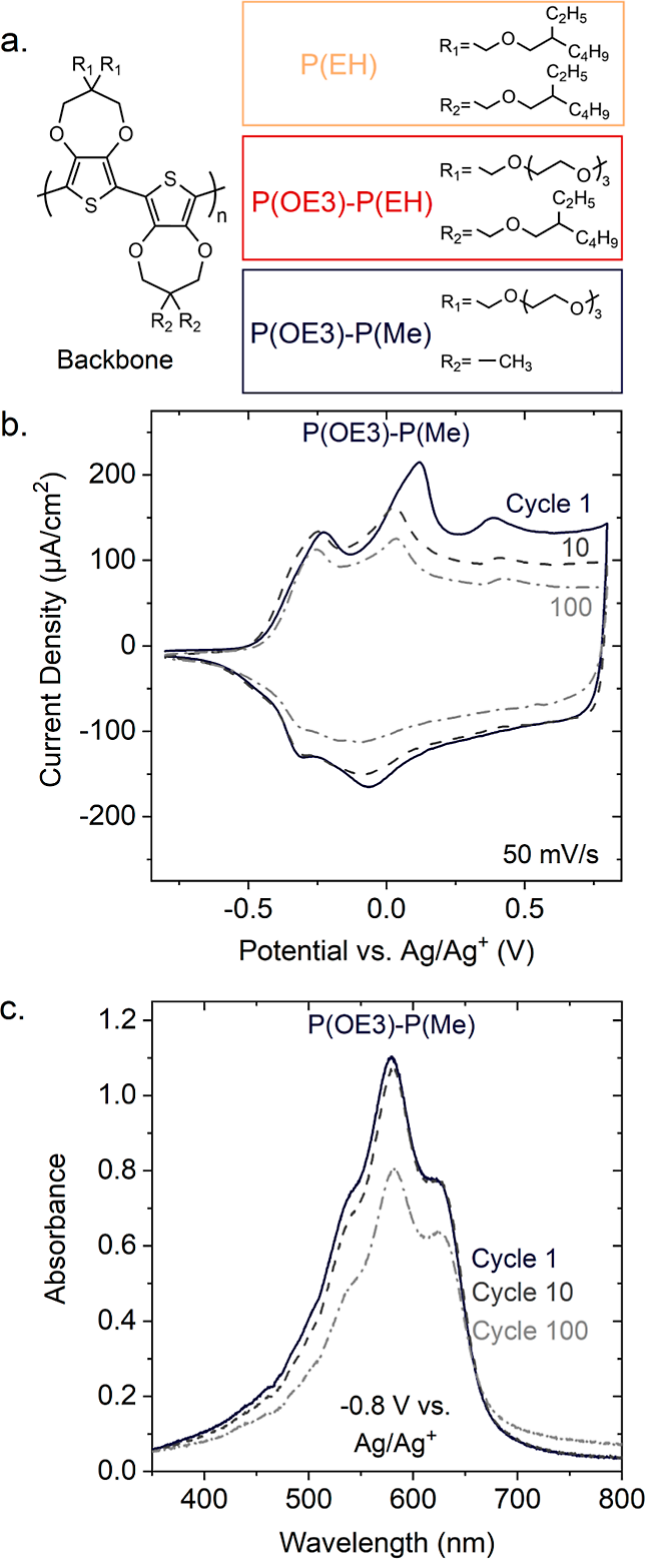
(a) Chemical structures of the poly(3,4-propylenedioxythiophene), P(ProDOT), derivatives investigated in this work. Left: P(ProDOT) backbone structure. Right: selected side-chain motifs. (b,c). Examples of durability issues in redox active polymers. (b) Cyclic voltammograms of P(OE3)-P(Me) during electrochemical cycling in 0.5 M tetrabutylammonium hexafluorophosphate (TBA^+^PF_6_^–^) dissolved in degassed propylene carbonate (PC), showing a decrease in the redox current upon repeated cycling. (c) Spectroelectrochemistry results for P(OE3)-P(Me) in the same electrolyte (0.5 M TBA^+^PF_6_^–^/PC) reveal a decrease in neutral-state linear absorbance upon repeated cycling, often referred to as “break-in”. Absorbance spectrum/cyclic voltammogram after one doping/dedoping cycle (solid lines); after 10 cycles (dark gray dashed lines); and after 100 cycles (light gray dash-dot lines).

The example of P(OE3)-P(Me) illustrates the challenge that persists in applying π-conjugated polymer semiconductors, including P(ProDOT)s, at large scale, as many important structure/property interrelations are still ill understood. Specifically, drastic differences in their electrochemical response are reported even for polymers of relatively similar chemical designs and/or when, e.g., changing the electrolyte.^8,11^ This renders the design of new materials an intricate task and limits our capability to predict properties from the outset.

Here, we selected, in addition to P(OE3)-P(Me), two chemically similar P(ProDOT)s to decipher which structural features dictate the electrochemical response of this class of polymers. Specifically, we chose poly(3,3-di(2,5,8,11-tetraoxadodecyl)-3′,3′-bis(((2-ethylhexyl)oxy)methyl)-3,3′,4,4′-tetrahydro-2H,2′H-6,6′-bithieno[3,4-*b*][1,4]dioxepine), P(OE3)-P(EH), an alternating copolymer with OE3 substituents in R_1_, as in P(OE3)-P(Me), but with 2-ethylhexyloxy, EH, side chains in R_2_; and poly(3,3-bis(((2-ethylhexyl)oxy)methyl)-3,4-dihydro-2H-thieno[3,4-*b*][1,4]dioxepine), P(EH), a homopolymer with apolar EH substituents in both the R_1_ and R_2_ positions (see chemical structures in Figure 1a). We chose this series of P(ProDOT)s as these three polymers can be expected to feature varying surface polarities due to the different side-chain substitutions and, therefore, a diverse range of swelling behaviors. In addition, all three polymers display little long-range order (Supporting Information), facilitating comparisons between the three systems. Synthetic details and procedures as well as purity and molecular-weight characterization information are reported in the Supporting Information (Schemes S1–S3 and Figures S1–S2).

We start our discussions with results obtained from contact-angle measurements using 0.5 M TBA^+^PF_6_^–^ in PC, applying drops of this commonly used polar aprotic organic electrolyte onto thin films of the three materials (see Figure S3). Our results indicate that the surface polarities are indeed different for the three materials (Figure 2a). The notable increase in contact angle from P(OE3)-P(Me) to P(OE3)-P(EH) implies that the surface polarity significantly decreases. Using EH-substituents in both R_1_ and R_2_, as in P(EH), has little effect compared to P(OE3)-P(EH), which is somewhat surprising considering the relatively apolar nature of the EH moieties.

**Figure 2:**
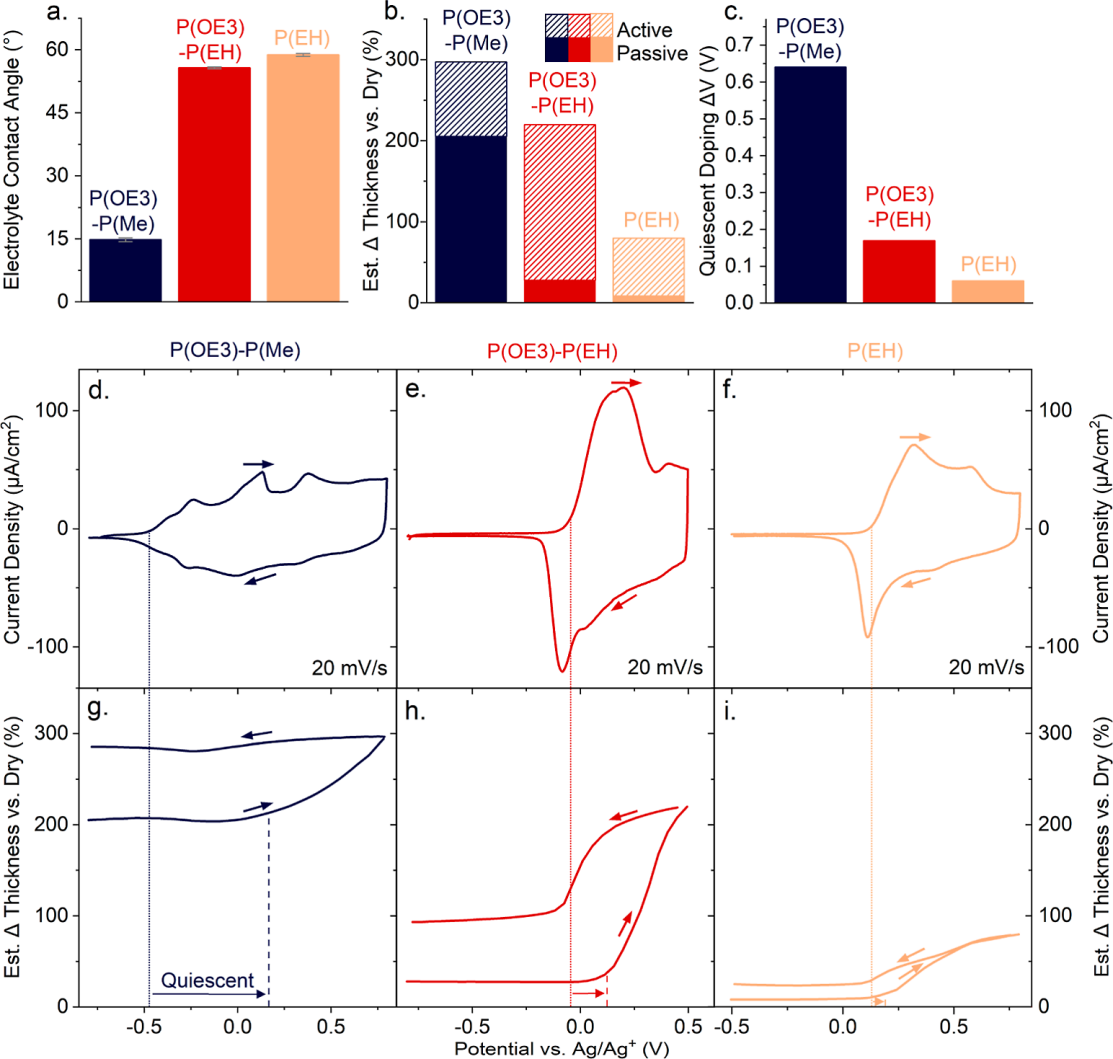
Swelling and electrochemical behavior of the selected P(ProDOT)s during the first electrochemical cycle. (a) Contact angles (*n* ≥ 4) of 0.5 M TBA^+^PF_6_^–^/PC droplets on thin films of the three selected P(ProDOT)s, revealing that P(OE3)-P(EH) and P(EH) are relatively apolar, while P(OE3)-P(Me) is polar. (b) Thickness change of swollen P(ProDOT) films relative to their dry state recorded during passive (solid bars) and active swelling (dashed bars). The latter values were estimated via the third overtone frequency shift at films’ maximum doping potentials, i.e., at 0.8 V for P(OE3)-P(Me) and P(EH) and at 0.5 V for P(OE3)-P(EH). (c) Extent of quiescent doping, i.e., the potential interval where oxidation is observed in the cyclic voltammogram without any associated thickness change, deduced from the electrochemical quartz-crystal microbalance with dissipation (EQCM-D) data shown in panels (d–i). (d–f) Cyclic voltammograms (first redox cycle) were recorded for P(OE3)-P(Me), P(OE3)-P(EH), and P(EH) at a scan rate of 20 mV/s. A detailed discussion on the different electrochemical behaviors of the three polymers is found in the Supporting Information (Figures S7–S8). (g–i) Estimated thickness changes measured during electrochemical cycling (first cycle) as estimated via the third overtone frequency shifts. Dotted lines in panels (d–i) indicate the onset potentials of oxidation, and the dashed lines in panels (g–i) indicate the onset of active-swelling; arrows in panels (g–i) highlight the quiescent doping interval (data used in panel c). Taking the first derivative of the thickness (see Figure S11 in the Supporting Information) assists identifying these critical potentials; it also helps visualizing the swelling/deswelling behavior of the three polymers.

Since it can be expected that the surface polarity of P(ProDOT) films will affect their swelling behavior, we went on and compared our contact-angle data with the passive swelling behavior, i.e., the *spontaneous* swelling of thin-film architectures upon exposure to an organic electrolyte.^12−14^ To characterize swelling, we performed quartz-crystal microbalance with dissipation monitoring (QCM-D) measurements (see the Supporting Information for details) and calculated the thickness change of polymer thin films between the as-cast dry state, and the passively swollen state upon exposure to 0.5 M TBA^+^PF_6_^–^/PC. By comparing the electrolyte-induced frequency- and dissipation-shifts for each polymer (Figures S4–S6), we find that the extent of passive swelling is around +205% (compared to the dry state) in P(OE3)-P(Me). Passive swelling decreases to about +30% in P(OE3)-P(EH) and +10% in P(EH) (Figure 2b, solid bars).

Collectively, the results summarized in Figure 2a,b imply a rather strong inverse correlation of the TBA^+^PF_6_^–^/PC contact angle with the three P(ProDOT)s’ passive swelling: the higher the contact angle, the lower the passive swelling. This suggests that the bulk property of passive swelling (Δthickness between dry state and films exposed to the electrolyte) can be directly linked to the material’s surface properties. Thereby, it is somewhat unexpected that P(OE3)-P(Me) (navy shading in Figure 2a,b) and P(OE3)-P(EH) (red shading; Figure 2a,b) exhibit notable differences in contact-angle and passive-swelling properties considering that both materials comprise polar OE3 side chains in R_1_ and apolar alkyl groups (Me and EH, respectively) in R_2_. Equally unexpected is that the contact-angle and passive-swelling response of the partially polar-substituted P(OE3)-P(EH) are comparable to those of P(EH) that features apolar groups in both R_1_ and R_2_.

Given that excessive swelling can lead to reduced stability (e.g., through mechanical film failure) and an overall decreased durability, especially in operando, we next assessed the “active” swelling of all three polymers.^15−17^ Active swelling of redox-active polymers is a critical physical process arising from the transport of charge-stabilizing counterions from the electrolyte, such as PF_6_^–^, through the active material under application of a bias,^14,18,19^ and is a requirement for successful electrochemical doping. After evaluating the potential dependence of electrochemical doping in the three polymers with cyclic voltammetry (Figure S7) and potential-dependent absorbance spectroscopy (Figure S8), we used electrochemical quartz crystal microbalance with dissipation monitoring (EQCM-D) to follow the active swelling process via simultaneous cyclic voltammetry measurements (Figure 2d–f) and in situ monitoring of real-time changes in thickness during electrochemical cycling (Figure 2g–i).^20^ The full data set of EQCM-D frequency and dissipation shifts are shown in Figure S9. A more detailed analysis of the redox properties of the three P(ProDOT)s can be found in the Supporting Information (Section 5).

Two observations can be immediately made from the data presented in Figure 2d–i. First, moving from P(OE3)-P(Me) to P(OE3)-P(EH) to P(EH), the onset of oxidation, as tracked by a change in current density, increases from −0.48, −0.05, and +0.13 V versus Ag/Ag^+^, respectively, as indicated with the dotted lines in Figure 2d–f. It may appear surprising that the three P(ProDOT)s have notably different electrochemical activity windows considering that they feature the same conjugated backbone chemistry. We note, though, that it has been previously shown that the redox potentials for these materials can strongly depend on the local backbone order (e.g., torsional order),^11,21^ which can be affected by the side-chain substituents.

Second, the EQCM-D data for the three polymers (see Figure 2d–i) indicate a strong difference in the potential interval where oxidation is observed without any associated thickness change—a phenomenon we will refer to here as “quiescent doping”. In Figure 2d–i, the quiescent doping regime is noted by the potential difference between the onset of oxidation given above (dotted lines) versus the onset of swelling (the potentials where an appreciable thickness change is recorded as ions are transported through the films to counterbalance charge generation) highlighted with dashed lines. Precisely, the onset potentials for active swelling are found at roughly +0.17, +0.12, and +0.19 V versus Ag/Ag^+^ for P(OE3)-P(Me), P(OE3)-P(EH), and P(EH), respectively. Clearly, for P(OE3)-P(Me) that displays very pronounced passive swelling, the quiescent doping regime is significant—over 650 mV—whereby initial film oxidation occurs without any active swelling and accumulation in carrier density primarily involves passively sorbed ions for charge balancing.^22^ Conversely, for P(OE3)-P(EH) and P(EH), the quiescent doping regime is essentially negligible because of the little passive swelling they undergo [note: electrochemical doping of the three polymers is also observed in spectroelectrochemistry, which provides a direct optical signature for the formation of polarons, independent of non-Faradaic charging events that can be present in cyclic voltammograms (ionic motion in the electrolyte). The potential to reach the doped state from the spectroelectrochemistry data is shown in Figure 3, black spectra; more details are given in Supporting Information Figure S8 that displays spectra taken at intermediate potentials].

**Figure 3:**
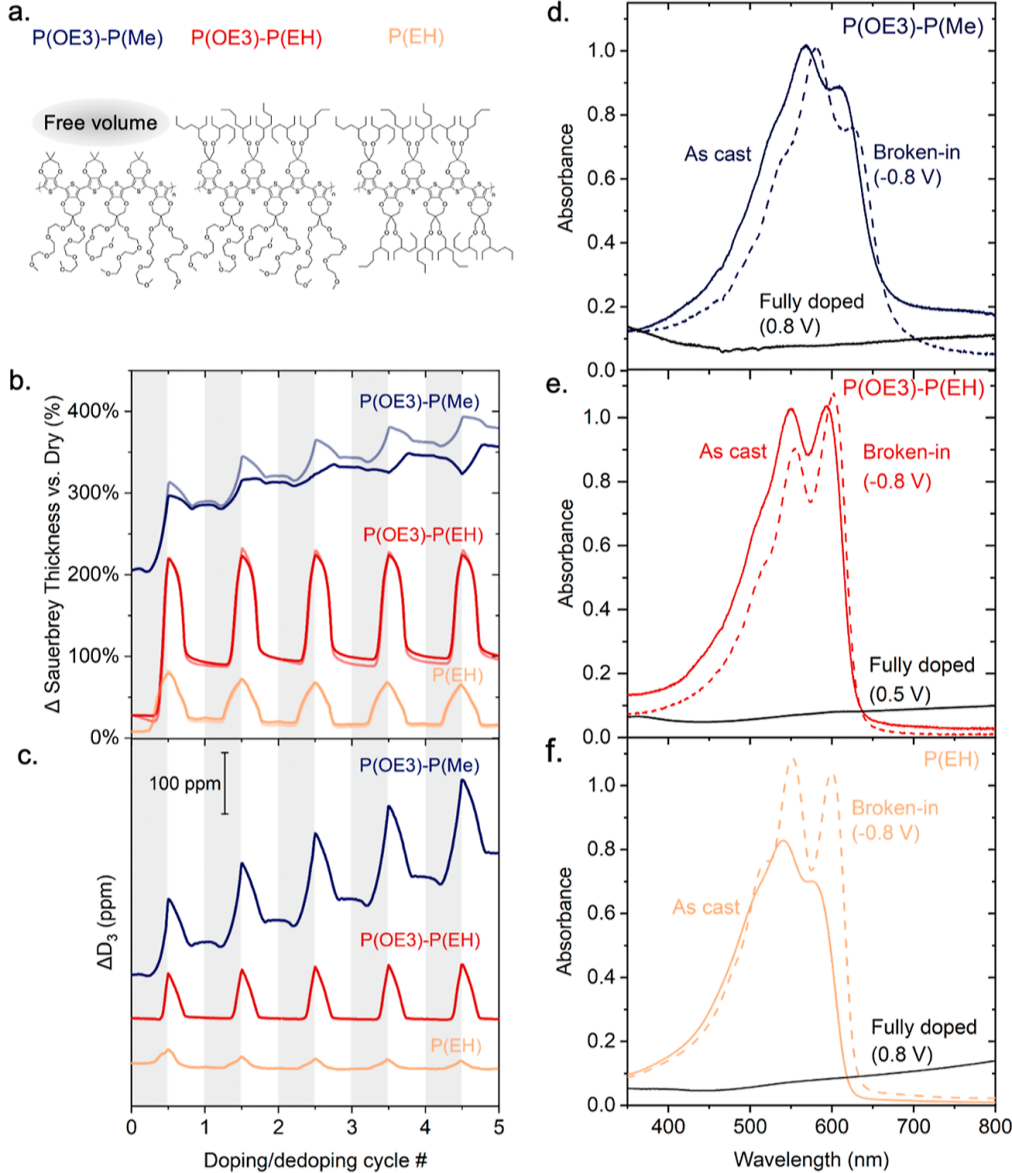
Multiple electrochemical cycles show that the side-chain free volume in P(ProDOT)s affects ion sorption/desorption and optical “break-in”. (a) Illustration of the difference in side-chain free volume that is provided by the three P(ProDOT)s studied here, i.e., of the void space provided locally (on the length scale of a monomer unit) depending on the side-chain motifs selected, rather than the free volume provided over larger scales, e.g., due to limited molecular packing. (b) Estimated change in film thickness recorded for P(OE3)-P(Me), P(OE3)-P(EH), and P(EH) over multiple cycles. Lighter traces show changes estimated from the fundamental frequency shift; darker traces show those from the third overtone frequency shift. (c) Third overtone dissipation shifts, measured relative to the passively swollen state, reveal changes in softness and thickness due to active swelling (Δ*D*_3_ data are offset and stacked for clarity). Note: the gray vs white shades in panels (e and f) differentiate the doping (gray) from the dedoping scans (white). (d–f) UV–vis absorption spectra of, respectively, P(OE3)-P(Me), P(OE3)-P(EH), and P(EH): pristine (colored, solid lines), electrochemically oxidized, i.e., fully doped (black, solid lines), and electrochemically reduced (colored, dashed lines); potentials are given with respect to an Ag/Ag^+^ reference electrode (calibrated vs Fc/Fc^+^, *E*_1/2_ = +90 mV). The less pronounced 0–0/0–1 vibronic peak ratios in pristine P(EH) and P(OE3)-P(Me) are indicative of more H- and/or HJ-like photophysical coupling compared to that of the electrochemically doped state [note: a detailed discussion of the absorption line shapes of P(EH) and P(OE3)-P(Me) can be found, respectively, in ref (11) and refs (7 and 21). Moreover, the switching potential for P(OE3)-P(EH) was chosen at 0.5 V vs Ag/Ag^+^ as we observed some irreversibility in the redox switching beyond 0.65 V. Specifically, the neutral spectrum could not be fully recovered. There was no sign of degradation but rather some residual absorbance above 700 nm, which points to incomplete electrochemical reduction of the doped form and charge trapping. Film thicknesses were chosen to yield a broken-in film with a peak neutral state absorbance of ≈1.0].

Further insights into active swelling were acquired by recording the thickness change measured between the undoped and doped states of the three materials (first redox cycle; Figure 2g–i). The obtained values, which provide the thickness change during active swelling, are summarized in Figure 2b as shaded bars overlaid onto the passive swelling data (filled bars). We find that P(OE3)-P(Me) experiences a total thickness change of close to +300% (with respect to the dry thickness) during active swelling to the maximum doping potential (0.8 V vs Ag/Ag^+^), compared to +200% versus dry thickness in passive swelling. Intriguingly, P(OE3)-P(EH) features a rather notable active-swelling thickness increase of close to +220% at the maximum doping potential, in stark contrast to this material’s modest passive swelling behavior—with a change in thickness of only around +30%. Active swelling in P(EH) results in a +80% total thickness change at the maximum doping potential after the first sweep, including the passive swelling prior to doping (ca. + 10%). Significantly, P(OE3)-P(Me) does not recover its initial thickness upon bias reversal to reach the neutral state, implying that the electrolyte is “trapped” in the material. For P(OE3)-P(EH) and P(EH), conversely, the thickness decreases upon application of a reverse bias—in case of P(EH) nearly to its initial state—from which we deduce that ions can be more readily desorbed after one oxidation/reduction cycle, though some electrolyte remains trapped in all three polymers.

The difference in the redox behavior of P(OE3)-P(Me) compared to the other two polymers becomes even more noticeable during cycling over five redox cycles (Figure 3) and indicates a substantial change in structure that occurs upon doping in this material. In fact, we observe that P(OE3)-P(Me) undergoes an increase in thickness of more than +350% compared to the initial dry film (navy-colored lines), which can be expected to eventually lead to mechanical failure. The simultaneous large increase in the dissipation factor (Figure 3c), which indicates an increase in softness (decrease in materials’ stiffness), supports the view that the thickness change is due to trapped propylene carbonate that plasticizes the material, as often observed in commodity polymers that comprise small molecular matter, such as trapped solvent.^23,24^ We do note that reversing the bias assists with some electrolyte solution desorption out of P(OE3)-P(Me), leading to a decrease in the dissipation factor during the reduction cycle and, thus, some recovery of the material’s stiffness. On the contrary, P(OE3)-P(EH) and P(EH) display high cyclability and reversibility (Figure 3b,c) with respect to film thickness and softness, as well as current density and neutral state optical density (Figure S10), although for both materials, we find that in the first redox cycle the neutral-state thickness is not fully recovered (Figure 2h,i).

Our set of observations can be explained by considering the local side-chain free volume, that is, the void space that may be provided on small length scales in the side-chain regions of a macromolecule depending on the side-chain motifs selected. In P(OE3)-P(Me), the alternating motif of OE3-side chains and the short (Me)-moieties at the R_2_ position result in a low local density, as schematically indicated in Figure 3a. Counterions can readily diffuse into this polymer, where they are “trapped” in this local free volume and cannot easily be released from the film even at application of a reverse bias (see Figure S11), similar to observations made on chemically doped polythiophenes, where specific side-chain motifs were found to open-up free volume, assisting dopant counterions to be accommodated near the polymer backbone.^25^ In the case in point here, i.e., P(OE3)-P(Me), “ion-trapping” likely is aided by the polarity and/or ionophilicity of the (OE3)-substituents and leads to an increase in the film’s thickness and softness after each redox cycle (Figure 3b,c, respectively). A pronounced irreversible behavior results from this, eventually contributing to the active material failure. Additionally, we emphasize that OE3 side chains clearly assist swelling (active and passive), yet they do not promote significantly faster doping kinetics in this electrolyte (see Figures S12 and S13). In contrast, for P(OE3)-P(EH), the EH-substituents on R2 seem to limit the free volume for “ion trapping”. Combined with their reduced ionophilicity, this results in a highly reversible cycling behavior, promising a durable performance, although we note that after the first redox cycle, the original thickness is not fully recovered. This suggests that a small portion of ions/electrolyte are irreversibly trapped in the material during this first redox cycle only, as mentioned above (see Figure 3b,c; full set of EQCM-D data are summarized in Figure S9). A similar behavior is observed for P(EH), emphasizing the beneficial effect of EH substituents for maintaining the film stability and integrity.

In conclusion, we have revealed that simple design criteria based on side-chain polarity cannot explain the complex redox behavior—or even the apparent surface polarity—of P(ProDOT)s and likely of other π-conjugated polymeric mixed conductors. Despite having similar chemical structures, P(OE3)-P(Me) and P(OE3)-P(EH) exhibit starkly different passive and active swelling behaviors. Especially for materials with a lack of pronounced long-range order (see Figures S14 and ref (25). for grazing incidence wide-angle X-ray scattering, and Figures S15 and S16 for fast scanning calorimetry data), a critical parameter that must be considered to understand these differences is the local free volume that is provided by certain side-chain motifs on (or below) the length scale of a monomer unit. A low side-chain free volume limits both passive swelling and undesired volume changes, thus improving cyclability and limiting mechanical failure. This local free volume stands in contrast to the more commonly discussed free volume found in the amorphous domains of a partly crystallizable polymer^26^ or, generally, less ordered regions of a material e.g., introduced by steric effects^4,11,27,28^ that can result in backbone disorder that hinders longer-range packing.

While free-volume is notoriously difficult to visualize for small quantities of materials such as those provided by thin films, we note that a low side-chain free volume may lead to a significant “break-in”, a feature often observed in the redox response, and sometimes also the UV–vis absorbance behavior, of polymer films, occurring during the first few doping cycles as the flux of ions/electrolyte through the film is equilibrated.^11,29,30^ Specifically, a low side-chain free volume, as given in P(EH), results in a drastic “break-in”, deduced from the pronounced changes recorded in the UV–vis absorption and electroactivity after the first redox cycles (Figure 3d–f), where transport of the PF_6_^–^ anion to counterbalance charging requires some displacement of the polymer backbone. Such a response is indicative of large conformational changes that accompany the electrochemical doping process as deduced from the fact that the neutral-state spectra of P(EH) after cycling display a much more structured line shape with a pronounced increase in the 0–0 transition. This observation can be attributed to a more J-aggregate-like character of the doped polymer compared to its as-cast neutral state.^31^ Likely, certain backbone segments of the polymer are planarized upon ion uptake into side-chain regions leading to a stronger intrachain coupling. In contrast, in P(OE3)-P(EH), ion uptake results in a less pronounced reordering of backbone segments leading to an electrochemical behavior with negligible “break-in” during cycling (see Figure 3e). Since ions/electrolytes are less trapped in P(OE3)-P(EH) compared to P(OE3)-P(ME), we infer that P(OE3)-P(EH) offers an excellent compromise with respect to side-chain free volume so that both undesired ion/electrolyte trapping and unwanted break-in effects are kept at an acceptable level. Our work, hence, delivers useful structure–property relations for mixed conducting polymers. Overall, it shows how a combination of complementary methodologies can be used to provide relevant comparisons between materials toward the design of low-swelling, durable soft mixed conductors for electrochemical applications.

## Experimental Section

### Polymer and Electrode Preparation

Briefly, the three polymers evaluated were synthesized via direct (hetero)arylation polymerization, with number-average molecular weights exceeding 10,000 g/mol, using previously reported conditions.^7,32^ Additional synthetic details for P(OE3)-P(EH) along with molecular weight and dispersity data for the three polymers can be found in the Supporting Information. Polymer films were processed onto the desired substrates via spray-coating from 4 mg/mL chloroform solutions using an Iwata Eclipse HP-BC airbrush to the desired thickness.

### Electrochemistry and Spectroelectrochemistry

Electrochemical characterization was performed in 0.5 M tetrabutylammonium hexafluorophosphate (TBAPF_6_) in [propylene carbonate (PC)]. The potential was controlled with either a PINE WaveNow potentiostat interfaced with AfterMath software (spectroelectrochemistry) or a Gamry Reference 3000 potentiostat interfaced with Microvacuum BioSense software (EQCM-D). All potentials are reported versus an Ag/Ag^+^ pseudoreference electrode (calibrated vs ferrocene/ferrocenium, *E*_1/2_ = 90 mV) and a platinum flag or wire coil counter electrode was used in all measurements. For the spectroscopic measurements, the films were deposited onto ITO-coated glass slides, and spectra were recorded with an Ocean Optics USB2000+ fiber-optic spectrophotometer or a Cary 5000 UV–vis–NIR spectrophotometer using a 1 cm path length quartz cuvette as a three-electrode cell. The doping kinetics were determined using chronoabsorptometry, where the absorbance at λ_max_ related to the π–π* was continuously monitored as the films switched between the neutral and fully doped sates for various pulse lengths (10–0.25 s).

### Swelling Studies

Passive swelling was determined with a Biolin Scientific QSense Analyzer multichannel quartz crystal microbalance with dissipation monitoring (QCM-D) capabilities, whereas potential-dependent (active) swelling experiments were performed with a Gamry Reference 3000 potentiostat coupled to a Gamry eQCM-I Mini. For these measurements, polymer films were deposited via spray casting to a thickness of 80–100 nm onto Au-coated Qsensors manufactured by Biolin Scientific. More details on the cleaning procedure, estimation of film thickness changes, and data processing can be found in the Supporting Information.

### Contact Angle Measurement

Contact angles were obtained from droplets of 0.5 M tetrabutylammonium hexafluorophosphate (TBA^+^PF_6_^–^) deposited on spray-coated thin films via measurement with a Ramé-Hart Standard Goniometer 250-00-115. Averages of the left and right contact angles were used for at least four droplets, as analyzed via DropIMAGE standard software. Contact angle images for 0.5 M TBA^+^PF_6_^–^ and DI water on the three P(ProDOT)s are available in the Supporting Information.

### Grazing Incidence Wide-Angle X-ray Scattering

X-ray scattering data for P(EH) were collected at Brookhaven National Laboratory’s National Synchrotron Light Source II (NSLS-II). A standard sample of silver behenate (AgBH) was also measured to determine the beam center and sample-detector distance. Patterns were captured from 13.5 keV X-ray beams with substrates positioned at an incidence angle of 0.14°. Raw WAXS data were analyzed using the Nika 2D SAS Macros available in Igor Pro 9.^33^ Grazing incidence wide-angle X-ray scattering (GIWAXS) data for O(OE3)-P(EH) and P(OE3)-P(Me) are reported in ref (25).

### Fast Scanning Calorimetry

Fast scanning calorimetry was conducted under nitrogen using a Mettler Toledo Flash DSC 1 equipped with a Huber TC100 cooler to control the temperature between −90 and +450 °C. Powder P(ProDOT) samples were deposited onto Mettler Toledo Standard MultiSTAR UFS 1 chips. The samples were scanned multiple times, aged and unaged, at a ramp rate of 4000 K/s as indicated with more detail in the right panel of Figure S15a (see the Supporting Information). The thermograms from aged and unaged samples were then compared to identify any distinct phase transitions induced by the differing thermal histories.

## Supporting Information Available

Materials synthesis and characterization procedures, experimental methods, and supplementary structural characterization data (PDF)
