# Supplementary material for: Role of Side-Chain Free Volume on the Electrochemical Behavior of Poly(propylenedioxythiophenes)
Source: Chem Mater. 2024 Mar 12;36(6):2634–41. doi: 10.1021/acs.chemmater.3c02122 (PMC10976628; doi:10.1021/acs.chemmater.3c02122)
Supplement: Supplementary file 1 — cm3c02122_si_001.pdf [file cm3c02122_si_001.pdf]

Supporting Information

The role of side-chain free volume on the electrochemical behavior of poly(propylenedioxythiophenes)

Marlow M. Durbin [a], Alex H. Balzer [a†], John R. Reynolds [b,c], Erin L. Ratcliff [d], Natalie Stingelin [a,b], Anna M. Österholm* [c]*

[a]School of Chemical and Biomolecular Engineering, Georgia Institute of Technology, Atlanta, GA 30332, United States

[b]School of Materials Science and Engineering, Georgia Institute of Technology, Atlanta, GA 30332, United States

[c]School of Chemistry and Biochemistry, Georgia Institute of Technology, Atlanta, GA 30332, United States

[d]Department of Chemical and Environmental Engineering, The University of Arizona, Tucson, AZ 85721-0012, United States

1. Materials

P(OE3)-P(Me), and P(EH) were synthesized via direct (hetero)arylation polymerization (DHAP), as previously reported.[1,2] The number average molecular weight $\overline{M_N}$ of P(OE3)-P(Me) was determined to be 27,000 g/mol (vs. polystyrene) with a dispersity Đ of 2.4. The number average molecular weight $\overline{M_N}$ of P(EH) was determined to be 11,700 g/mol with a dispersity Đ of 1.7. The molecular weight characterizations were performed with stabilized chloroform as the eluent at 40

°C using a Tosoh EcoSEC equipped with a 6 mm x 15 cm, 3 and 5 μm TSKgel SuperHZM-M column and a refractive index detector. The synthesis and characterization of P(OE3)-P(EH) is described in sections 1a-1c below.

1a. P(OE3)-P(EH) – monomer synthesis and characterization

Dimethoxythiophene (98%, Oxchem), pivalic acid (99%, Sigma), $Pd(OAc)_2$ (98%, Strem Chemicals), K_2CO_3 (anhydrous, Oakwood Products), 18-Crown-6 (99%, Acros Organics), 2-ethylhexanol (≥ 99.0%, Sigma Aldrich), 2,2-bis(bromomethyl)propane-1,3-diol (42.1 g, 0.16 mol), and diethyldithiocarbamic acid diethylammonium salt (97%, TCI America) were all used as received. Anhydrous N,N-dimethylformamide (DMF) was purchased from EMD and was also used as received. Dimethylacetamide (DMAc, HPLC grade, Alfa Aesar) was filtered prior to use. Methanol, acetone, toluene, and chloroform were purchased from Fisher Scientific and used without further purification. Hexanes were purchased from VWR and used as received. 2,5-dibromo-3,4-(2-ethylhexyloxy)-thiophene was synthesized according to published methods and confirmed by ^{1}H-NMR.[3]

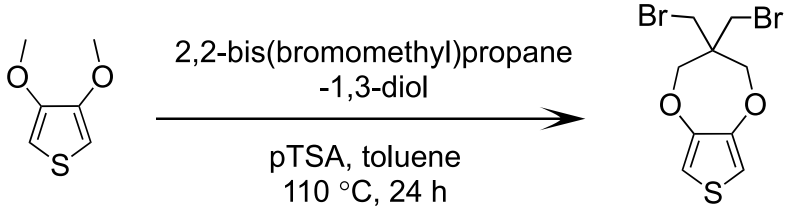

Scheme S1. Synthesis of 3,3-bis(bromomethyl)-3,4-dihydro-2H-thieno[3,4-b][1,4]dioxepine.

Dimethoxythiophene (21.1 g, 0.15 mol) and 2,2-bis(bromomethyl)propane-1,3-diol (42.1 g, 0.16 mol) were added into anhydrous toluene (600 mL) in a 1,000 mL round bottom flask equipped

with magnetic stir bar. The solution was premixed for 10 minutes before slowly adding *p*-toluenesulfonic acid (pTSA, 2.78 g, 0.015 mol). The reaction was refluxed for 24 hours, after which the reaction was quenched with 175 mL of deionized (DI) water. The organic layer was extracted three times into diethyl ether then passed over a silica plug using 4:1 hexanes:dichloromethane (DCM) and evaporated to yield 42 g of white solid (yield: 85%) . ^{1}H NMR (300 MHz, $CDCl_3$) δ(ppm) 6.50 (s, 2H), 4.10 (s, 4H), 3.61 (s, 4H). ^{13}C NMR (75 MHz, $CDCl_3$) δ(ppm) 148.62, 105.73, 74.10, 46.20, 34.40.

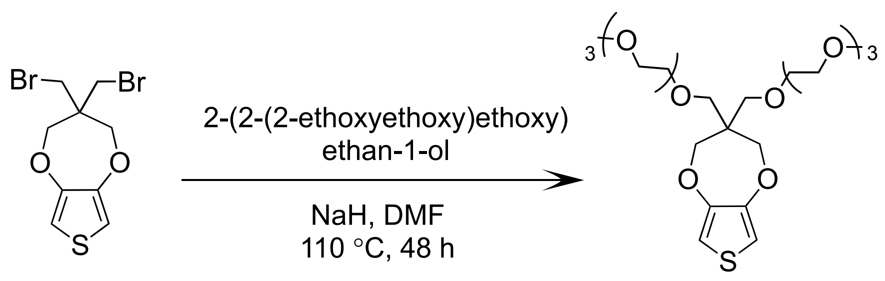

Scheme S2. Synthesis of 3,3-di(2,5,8,11-tetraoxadodecyl)-3,4-dihydro-2H-thieno[3,4-b][1,4]dioxepine.

3-bis(bromomethyl)-3,4-dihydro-2H-thieno[3,4-b][1,4]dioxepine (6.7 g, 60 mmol), 2-(2-(2-ethoxyethoxy)ethoxy)ethan-1-ol (10.0 g, 0.561 mol), and 400 mL of anhydrous DMF were added to a dry 1,000 mL round bottom flask equipped with a magnetic stir bar under argon. NaH (3.71 g, 57-63%) was added slowly to the reaction mixture while stirring. After the hydride was fully dissolved, the reaction was heated at 110 °C for two days. The reaction mixture was cooled to room temperature after which the organic layer was extracted three times with DCM and brine before being dried with $MgSO_4$. Finally, the mixture was filtered to remove $MgSO_4$. The solvent was concentrated, and the crude product was purified by silica gel chromatography using DCM to obtain 6.0 g of a clear, slightly yellow oil (yield: 60%).

1b. P(OE3)-P(EH) polymerization

After monomer synthesis and purification, the poly(propylenedioxythiophene) copolymer P(OE3)-P(EH) was synthesized via Pd-catalyzed direct hetero(arylation) polymerization shown in Scheme 1, and with procedure summarized below.

.

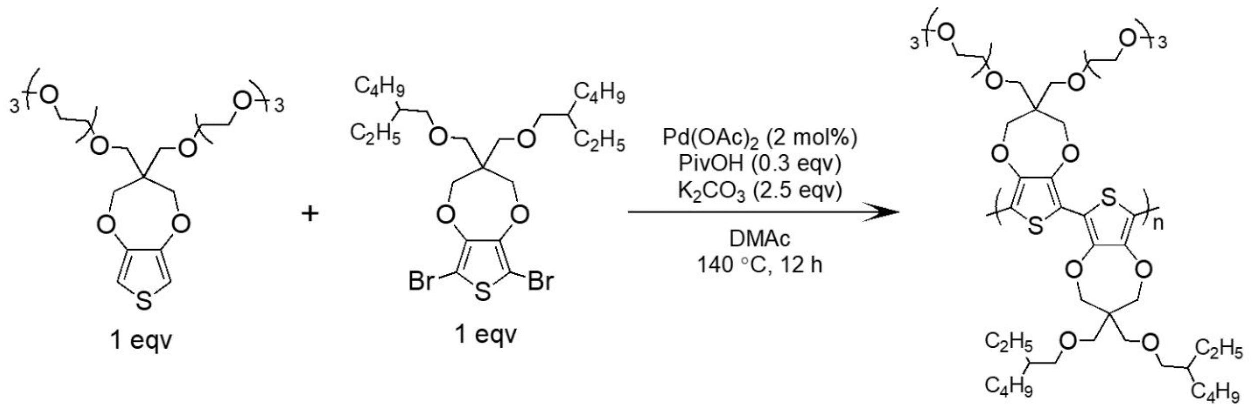

Scheme S3. Polymerization mechanism of P(OE3)-P(EH).

1.0 equivalent of both co-monomers, pivalic acid (0.3 equiv.), potassium carbonate (2.5 equiv.), and a stir bar were added to a dried and evacuated Schlenk flask at room temperature. Reagents were dissolved by addition of anhydrous DMAc. The flask was subsequently sealed and purged for 10 minutes while stirring. The catalyst palladium(II)acetate ($Pd(OAc)_2$, 2 mol %) was added

to the reaction mixture, under a blanket of argon, and then lowered into a 140 ˚C oil bath. The reaction was allowed to stir for twelve hours under positive argon pressure. The polymer was then precipitated into methanol and filtered into a soxhlet extraction thimble. Washes were performed using methanol, acetone, hexanes, and finally chloroform to dissolve the P(OE3)-P(EH). Approximately 20 mg of a palladium scavenger (diethylammonium diethyldithiocarbamate) and ≈20 mg of 18-crown-6 were added to the chloroform fraction, which was then allowed to stir for two hours at 50 °C. Chloroform was subsequently evaporated via rotary evaporation until 3 mL of polymer solution remained in the flask. The polymer was finally precipitated in ≈300 mL of methanol. The precipitate was vacuum filtered using a nylon pad (with a pore size of 20 μm) and washed with a large volume of methanol before drying. The dried material was collected into a vial and dried under vacuum for two days. Product purity was verified by ^{1}H-NMR and elemental analysis. ^{1}H NMR spectra for the polymers were collected using the Bruker Corporation DRX 700 MHz spectrometer. The chemical shift was set based on residual $CHCl_3$ (in the $CDCl_3$ solvent, 25 °C) as an internal standard set to 7.26 ppm (see Figure S1). The number-average molecular weight and dispersity were determined via gel permeation chromatography (Tosoh EcoSEC equipped with a RI detector) to be M_n = 41,700 g/mol and Đ = 1.9 versus PS in $CHCl_3$ at 40 °C (see Figure S2). ^{1}H NMR (700 MHz, CDCl3, 25 ˚C) δ 4.20 (br, 8H), 3.74-3.50 (m, 34H), 3.34-3.34 (m, 8H), 1.54

(br, 2H), 1.45-1.23 (m, 16H), 0.93-0.87 (m, 12H). Anal. calcd.: C 60.99, H 8.53, S 6.78, Found: C 60.99, H 8.50, S 6.84.

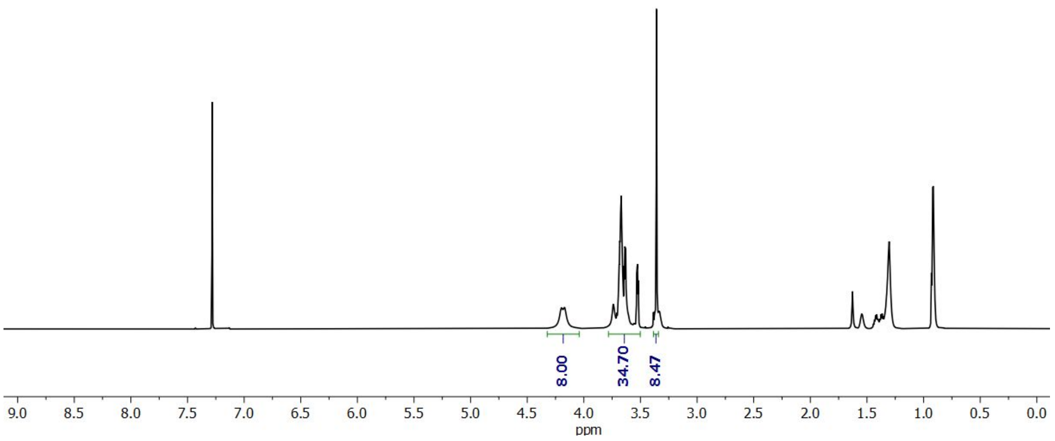

Figure S1. ^{1}H-NMR (700 MHz) spectrum and peak integration of P(OE3)-P(EH) in $CDCl_3$.

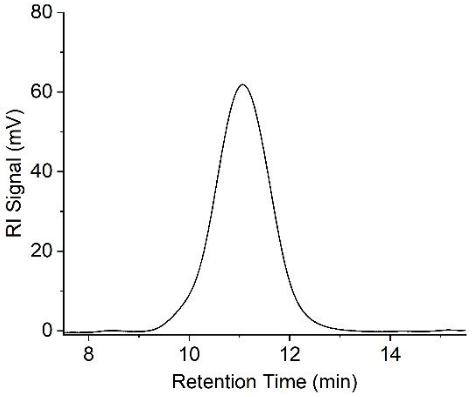

Figure S2. Gel permeation chromatogram of polymer P(OE3)-P(EH) in $CHCl_3$ at 40° C. M_n = 41,700 g/mol, Đ = 1.9 vs. poly(styrene).

2. Thin-film preparation

Polymer films were processed via spray-coating from 4 mg/mL chloroform solutions onto various substrates using an Iwata Eclipse HP-BC airbrush with an optimized argon pressure of approximately 20 psi to deposit polymer thin films to the desired thickness. An average dry film thickness of ≈90 nm was chosen to maximize the quality of the QCM-D response observed while minimizing the relative contribution of roughness to the film's swelling effects. For the spectroelectrochemical measurements the polymer films were deposited onto ITO/glass slides (Delta Technologies Ltd., 25 x 75 x 0.7 mm^3, sheet resistance 8-12 Ω/sq, cleaned by sonication in toluene, acetone, and isopropanol). For the QCM-D and EQCM-D measurements the polymers were coated onto 5 MHz Au-coated Q-Sense quartz sensors (QSX 301) from Biolin Scientific.

After coating, dry film thicknesses were measured via stylus profilometry, using a Bruker Dektak profilometer and averaging over six to eight locations on each film.

3. Contact angle goniometry

Contact angle measurements were obtained using a Ramé-Hart Standard Goniometer 250-00-115, with the data analysis done on the DropIMAGE standard software using averages of the left and right contact angles for at least four droplets (n ≥ 4) of DI water and 0.5 M $TBA^+PF_6^-$/PC.

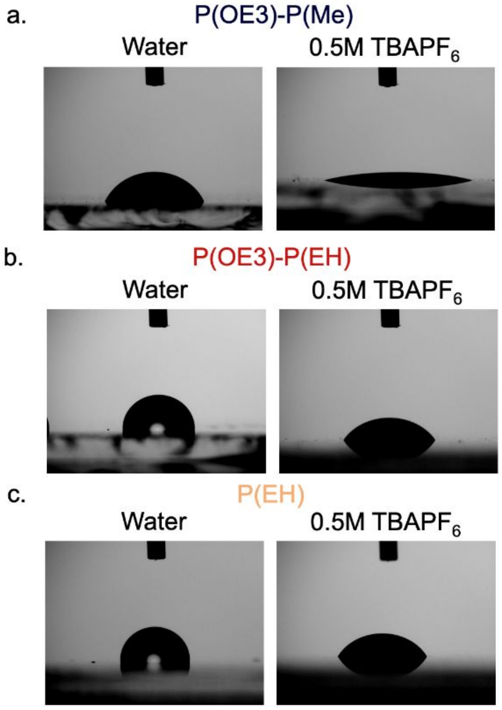

Figure S3. Deionized (DI) water and 0.5 M TBA^+PF6^-/PC contact angle images for **a.** P(OE3)-P(Me), **b.** P(OE3)-P(EH), and **c.** P(EH).

4. Passive swelling characterization

To estimate mass changes due to electrolyte uptake in the absence of bias (passive swelling), a Biolin Scientific QSense Analyzer multichannel quartz crystal microbalance with dissipation monitoring (QCM-D) was used. Au-coated Qsensors manufactured by Biolin Scientific were cleaned with a sodium dodecyl sulfate (SDS) solution, DI water, and isopropyl alcohol prior to drying with argon gas and exposure to 10 minutes of UV-ozone surface treatment. Polymer films were then deposited on the QSensors adhered to glass via spray coating as described in section 2. Stylus profilometry was conducted on 6-8 locations of the surrounding glass substrate to estimate the thickness of the dry film on the QSensor.

Three polymer-coated Qsensors and one bare reference Qsensor were mounted into the Qsense Analyzer. Chip resonance was found with an air overlayer and monitored for five minutes, prior to introduction of the 0.5 M $TBA^+PF_6^-$/PC electrolyte solution. The electrolyte solution was pumped through the QCM-D flow cells using an IPC high-precision multichannel peristaltic pump (ISMATEC, IDEX, USA) at a flow rate of 0.1 mL/min. Frequency (Figure S4) and dissipation

(Figure S5) shifts of the four Qsensors were monitored on the 3rd through 9th overtones for an additional 60 minutes after introduction of liquid electrolyte. Note: the three P(ProDOT)s are completely insoluble in common electrolytes such as acetonitrile, water as well as the $TBA^+PF_6^-$/PC electrolyte solution used here (cf. Ref. 4, which shows that P(EH) can withstand 200,000 redox cycles in an organic electrolyte).[4]

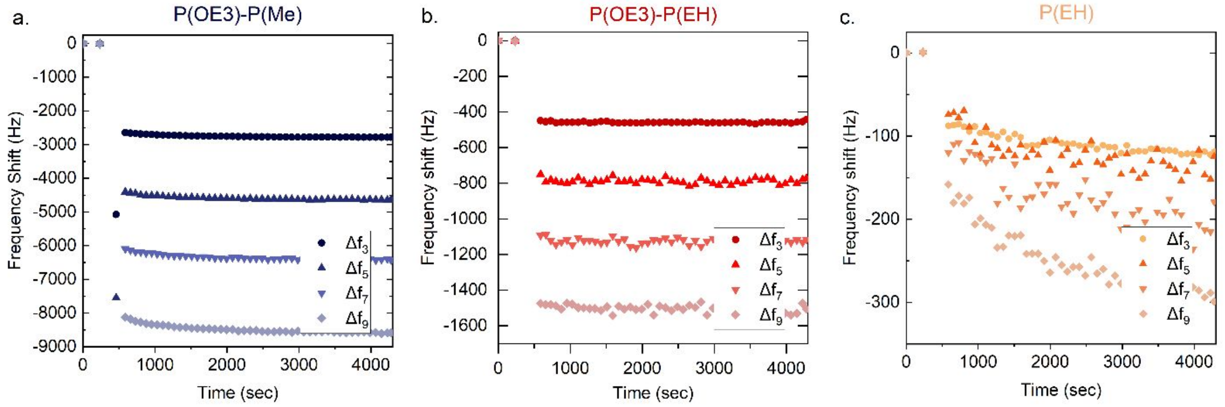

Figure S4. Frequency shifts for all polymers swelling during exposure to 0.5 M TBA^+PF6^-/PC (bulk fluid shift removed). Film frequencies for 3rd-9th overtones recorded in air and 0.5 M TBA^+PF6^-/PC overlayers, with bulk fluid shift removed. Dry film thicknesses (*via* profilometry) were 80, 97, and 87 nm for **a.** P(OE3)-P(Me), **b.** P(OE3)-P(EH), and **c.** P(EH), respectively. 1 in 50 data points plotted for clarity.

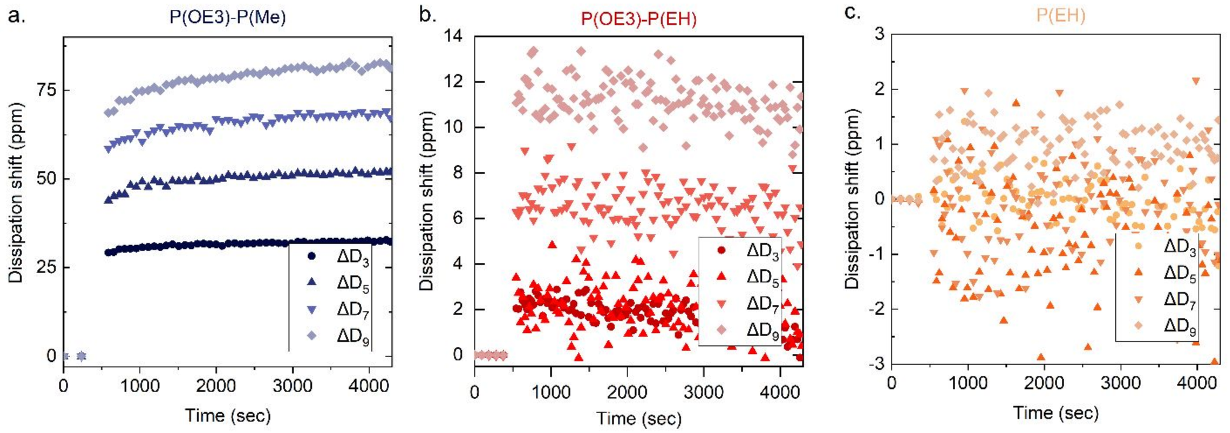

Figure S5. Dissipation shifts for all polymers swelling during exposure to 0.5 M TBA^+PF6^-/PC (bulk fluid shift removed). Film dissipations for 3rd-9th overtones recorded in air and 0.5 M TBA^+PF6^-/PC overlayers, with bulk fluid shift removed. Measurements performed on spray-coated films with dry thicknesses (*via* profilometry) of **a.** ≈80 nm P(OE3)-P(Me), **b.** ≈97 nm P(OE3)- P(EH), and **c.** ≈87 nm P(EH). 1 in 50 data points plotted for clarity.

Film thickness changes due to passive electrolyte uptake (passive swelling) were then calculated as follows: given a constant film thickness t across the circular QSensor active sensing area A, the dry and swollen film masses are given by $m_{\mathbf{dry}} = \rho_{\mathbf{dry}} t_{\mathbf{dry}} A$ and $m_{\mathbf{swollen}} = \rho_{\mathbf{swollen}} t_{\mathbf{swollen}} A$, respectively, where dry film thicknesses were measured via profilometry. The mass change attributed to swelling Δm is thus:

$$\Delta m = m_{\text{swollen}} - m_{\text{dry}} = A\rho_{\text{swollen}} t_{\text{swollen}} - A\rho_{\text{dry}} t_{\text{dry}} \quad \text{(Eq. 1)}$$

The Sauerbrey equation relates mass change per unit area on the QSensor to the frequency shift of the coated sensor attributed to the swelling process, $\Delta f_{\text{swelling}}$, normalized by the overtone number n, for a sufficiently thin and rigid overlayer[5] (the reasonability of this approximation is discussed in detail in section 8).

$$\frac{\Delta m}{A} = -17.7 * \frac{\Delta f_{\text{swelling}}}{n} \quad [\text{ng/cm}^2] \quad \text{(Eq. 2)}$$

In three of the flow channels of the QSense Analyzer, the 3rd-9th overtone frequencies of the polymer-coated QCM-D sensors were simultaneously measured before and after the introduction of the same electrolyte by peristaltic pump. The final frequencies (of the wet, polymer-coated sensor) were then subtracted from the initial frequencies (of the dry, polymer-coated sensor) to calculate $\Delta f_{\text{air}\rightarrow\text{electrolyte, coated}}$, which we refer to here as the "total frequency shift".

A fourth flow channel of the QSense Analyzer was used to estimate the frequency shift attributed to the viscous damping generated by the liquid electrolyte overlayer. In this channel, the 3rd-9th overtone frequencies of a bare reference QCM-D sensor were measured before and after the introduction of electrolyte via peristaltic pump. The final frequencies (of the wet bare sensor) used alongside the initial frequencies (of the dry bare sensor) to calculate $\Delta f_{\text{air}\rightarrow\text{electrolyte, bare}}$, also known as the "bulk fluid shift". This shift is caused by the increased viscosity and density of the liquid electrolyte overlayer on the wet sensor, compared to the air overlayer on the dry sensor.[6,7]

The bulk fluid shift measured on the bare QSensor, $\Delta f_{\text{air}\rightarrow\text{electrolyte, bare}}$, was then subtracted from the total frequency shift of the polymer-coated QCM-D sensors, $\Delta f_{\text{air}\rightarrow\text{electrolyte, coated}}$, as shown in Equation 3. Note that the initial frequencies of each dry sensor were already subtracted in the steps above to account for dry sensor mass differences. The resulting frequency shifts are thus

attributed to the sensor mass changes caused by swelling of the pre-coated polymer layer, $\Delta f_{\text{swelling}}$. These $\Delta f_{\text{swelling}}$ values are displayed in Figure S4.

$$\Delta f_{\text{swelling}} = \Delta f_{\text{air}\rightarrow\text{electrolyte, coated}} - \Delta f_{\text{air}\rightarrow\text{electrolyte, bare}} \text{ (Eq. 3)}$$

Assuming the film remains pinned to the sensor surface, and thus its area A remains constant, we can rearrange equation 1 to show that the mass change must result from changes to film thickness and/or density:

$$\rho_{\text{swollen}} t_{\text{swollen}} - \rho_{\text{f, dry}} t_{\text{dry}} = \frac{\Delta m}{A} \text{ (Eq. 4)}$$

For analytical simplicity (see note below), the dry and swollen film’s densities are approximated as 1 g/cm^3. Therefore, after performing necessary unit conversions, the added thickness, and the corresponding percent change of the film thickness during passive swelling are calculated from combining equations 2 and 4:

$$t_{\text{swollen,}} - t_{\text{dry}} \cong -0.177 * \frac{\Delta f_{\text{swelling}}}{n} \text{ [nm] (Eq. 5)}$$

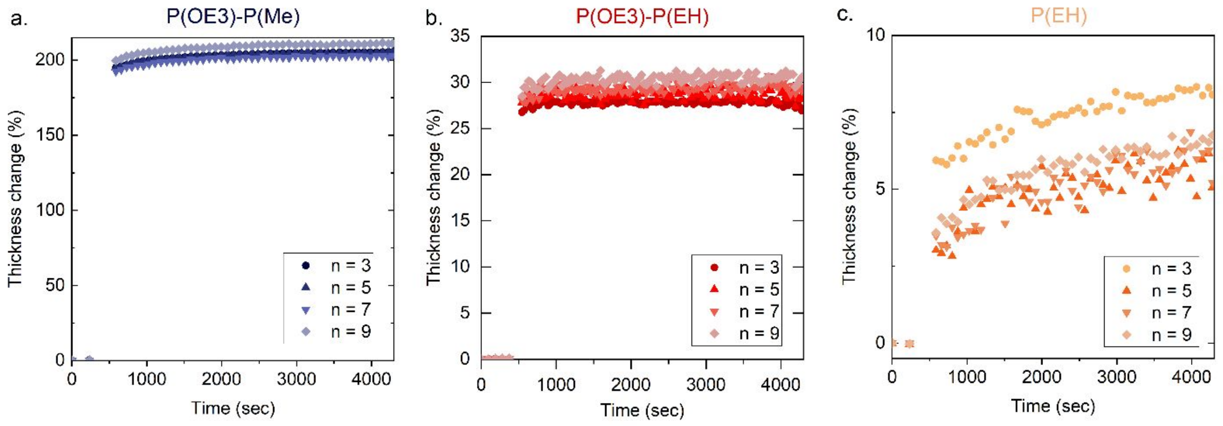

Figure S6. Sauerbrey thickness change calculated for 3rd-9th overtones for all polymers during passive swelling in 0.5 M $TBA^+PF_6^-$/PC. % Thickness changes relative to dry film thicknesses (measured via profilometry) for **a.** ≈80 nm P(OE3)-P(Me), **b.** ≈97 nm P(OE3)- P(EH), and **c.** ≈87 nm P(EH). 1 in 50 data points plotted for clarity.

We note that for systems that undergo significant swelling, such as, P(OE3)-P(Me), the assumption that the film density remains 1 g/cm^3 during the swelling process represents a simplified picture.

5. Electrochemical characterization

All electrochemical characterization was performed in 0.5 M tetrabutylammonium hexafluorophosphate ($TBA^+PF_6^-$, 98% AlfaAesar, purified by recrystallization from hot ethanol) in propylene carbonate (PC, 99.5 %, Acros Organics, was passed through a solvent purification system from Vac Atmospheres before use). PC was chosen as an advantageous low-volatility

carbonate, a family of solvents which is commonly used in Li-ion batteries. Tetrabutylammonium was chosen as a cation to avoid possible alkali ion chelation to oligoether side chains, a process that could influence redox switching. The redox behavior of the three polymers was evaluated in 0.5 M $TBA^+PF_6^-$/PC using cyclic voltammetry and potential stepping (i.e., bulk electrolysis). The potential was controlled with either a PINE WaveNow potentiostat interfaced with AfterMath software (for spectroelectrochemistry) or a Gamry Reference 3000 potentiostat interfaced with Microvacuum BioSense software (for EQCM-D). All potentials are reported versus an Ag/Ag^+ pseudoreference electrode (inner solution: 10 mM $AgNO_3$ dissolved in 0.5 M $TBA^+PF_6^-$ in acetonitrile) calibrated versus ferrocene/ferrocenium redox couple in 0.5 M $TBA^+PF_6^-$/PC. The ferrocene/ferrocenium redox couple $E_{1/2}$ was calculated as 90 mV by averaging the potentials of the anodic and cathodic current peaks. A platinum flag or wire coil counter electrode was used in all measurements.

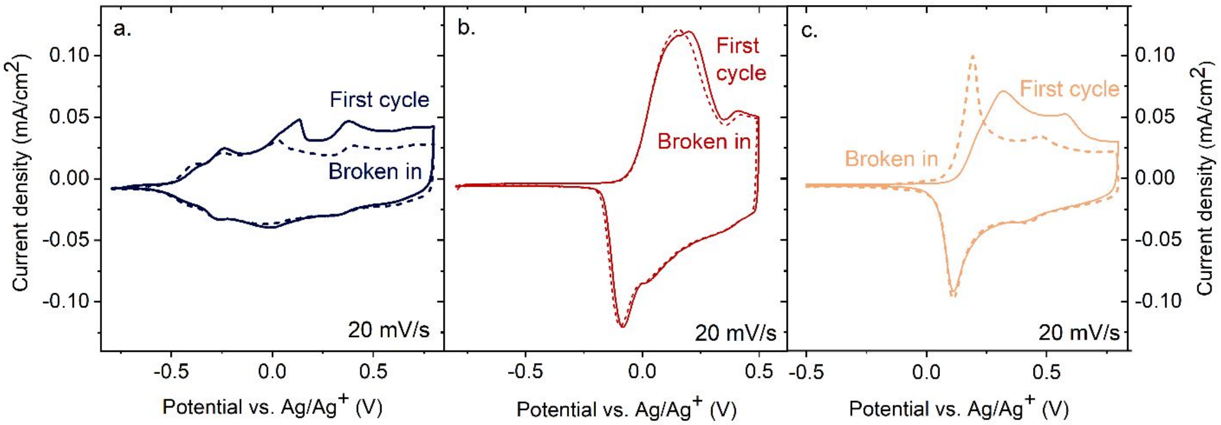

Figure S7. First (solid lines) and fifth (dashed lines) cyclic voltammograms of **a.** P(OE3)-P(Me), **b.** P(OE3)-P(EH), and **c.** P(EH) in 0.5 M $TBA^+PF_6^-$/PC at a scan rate of 20 mV/s.

The cyclic voltammograms of the three polymers above in Figure S7 are briefly discussed below, using insights obtained from spectroelectrochemistry (shown later in Figure S8). P(OE3)-P(Me) has the lowest onset potential during the first cycle of the three polymers, at -0.51 V vs. Ag/Ag^+. In addition to the initial oxidation peak, the material undergoes four other oxidation processes with onset potentials at -0.32 V, -0.06 V, 0.28 V, and 0.61 V vs. Ag/Ag^+ on the first scan. We attribute these peaks to progressive oxidation of neutral and doped P(OE3)-P(Me) sub-populations with distinct energetics due to differing local order/aggregation states, as suggested by the broken-in film's spectroelectrochemical behavior. Clearly, multiple sub-populations in the neutral and doped states are accessed, as suggested by the changing vibronic features as well as charge carrier absorption bands at increasing potentials.

P(OE3)-P(EH) has a higher oxidation onset potential at -0.07 V vs. Ag/Ag^+ during the first cycle. In addition to the initial oxidation feature, the material undergoes another distinct oxidation process with an onset potential of 0.36 V vs. Ag/Ag^+. We attribute these peaks to oxidation of two

P(OE3)-P(EH) sub-populations with distinct energetics due to differing local order/aggregation states found in spectroelectrochemistry. Specifically, the spectra show two neutral state sub-populations with distinct energetics due to differing local order/aggregation states, as deduced from the weakening of neutral state vibronic features between the onset of doping for the broken-in film at -0.1 V and the partially doped state at 0.1 V (see Figure S8). P(EH) has the highest first cycle oxidation onset potential of the three polymers, at 0.12 V vs. Ag/Ag^{+}. It undergoes a second oxidation process with an onset potential of 0.55 V vs. Ag/Ag^{+}. We attribute these peaks to oxidation of two P(EH) sub-populations with distinct energetics due to differing local order/aggregation states (see Figure S8). Indeed, spectroelectrochemistry reveals two neutral state sub-populations with distinct energetics due to differing local order/aggregation states, as suggested by the weakening of neutral state vibronic features between the onset of doping for the broken-in film at -0.1 V and the partially doped state at 0.2 V.

6. UV-Vis-NIR absorption spectroscopy

In situ spectroscopic analyses were performed on polymer films spray coated onto transparent conductive indium tin oxide (ITO) substrates supported on glass. Spectra were taken with an Ocean Optics USB2000+ fiber-optic spectrophotometer (Figure 3a-c) or a Cary 5000 UV-Vis-NIR commercial UV–Vis spectrophotometer (Figure S8) using a 1 cm path length quartz cuvette as a three-electrode cell. Bare ITO-coated glass slides were used as reference to remove the background absorption of the glass substrate from all measured spectra.

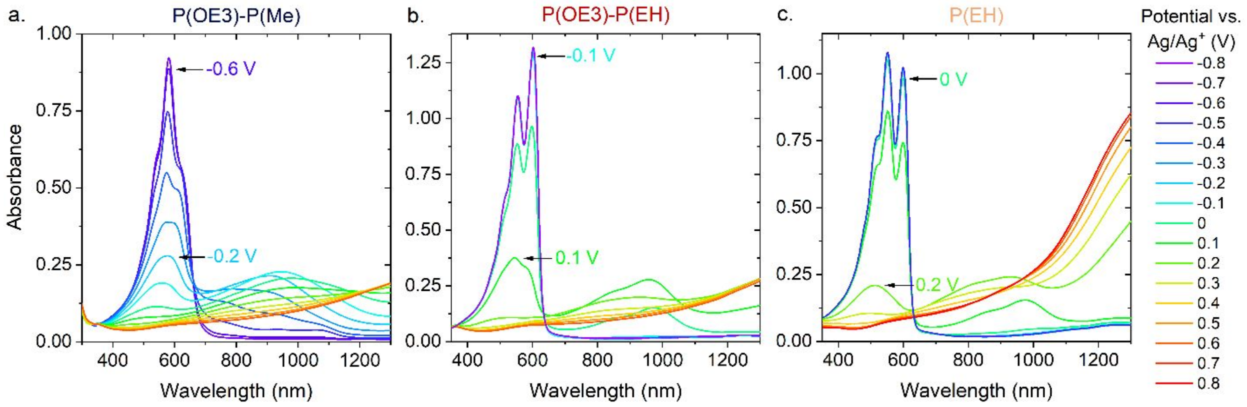

Figure S8. Full potential-dependent UV-Vis-NIR absorbance spectra for **a.** P(OE3)-P(Me), **b.** P(OE3)-P(EH), and **c.** P(EH) in 0.5 M $TBA^{+}PF_6^{-}$/PC vs. Ag/Ag^{+}.

7. Active swelling characterization

To estimate the mass changes due to electrolyte uptake with application of bias (active swelling), the potential was controlled via a Gamry Reference 3000 potentiostat coupled to a Gamry eQCM-I Mini with electrolyte and QCM-D sensor loaded in the supplied EQCM-I static cell (Microvacuum Ltd.). Frequency and dissipation shifts were collected on various overtones for P(OE3)-P(Me), P(OE3)-P(EH), and P(EH) (Figure S9). The mass changes associated with active swelling were then calculated using the same Sauerbrey thickness relation used for passive swelling measurements. Total thickness changes of the films (passive + active swelling) were then estimated by combining the thickness change due to active swelling with the above estimated thickness changes due to passive swelling.

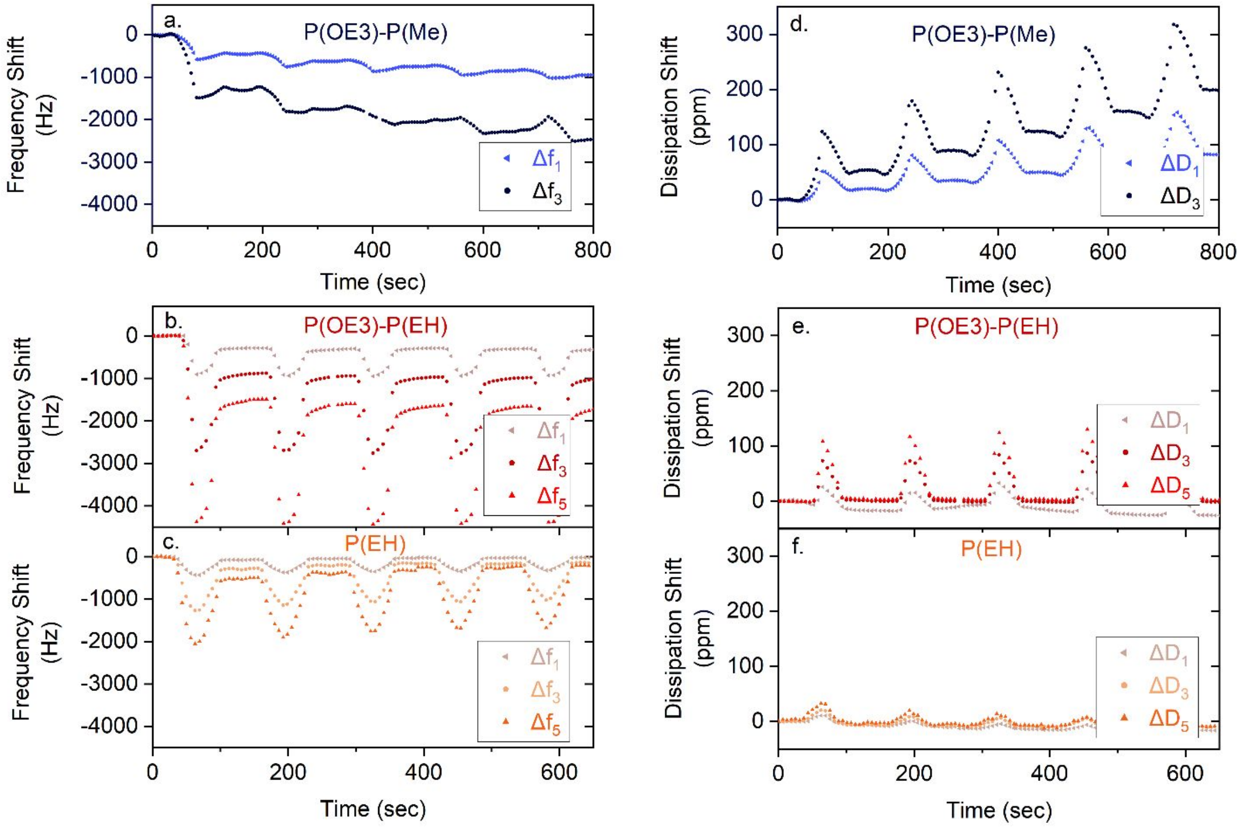

Figure S9. Raw EQCM-D active swelling frequency and dissipation shifts for all polymers in 0.5 M $TBA^+PF_6^-$/PC. **a.** Frequency response vs. time for P(OE3)-P(Me). Films cycled a scan rate of 20 mV/s from -0.8 V to 0.8 V (vs. Ag/Ag^+) for five cycles. Dry film had an average thickness of ≈96 nm. **b.** Frequency response vs. time for P(OE3)-P(EH). Films cycled a scan rate of 20 mV/s from -0.8 V to 0.5 V (vs. Ag/Ag^+) for five cycles. Dry film had an average thickness of ≈83 nm. **c**. Frequency response vs. time for P(EH). Films cycled a scan rate of 20 mV/s from -0.5 V to 0.8 V (vs. Ag/Ag^+) for five cycles. Dry film had an average thickness of ≈107 nm. **d.-f.** Dissipation factor shifts recorded for the same films as in panels a.-c., respectively. For clarity, only 1 out of 3 data points are plotted.

8. Cycling stability of P(OE3)-P(EH) and P(EH)

Analogous to the data presented in Figure 1, panels b and c, below are shown the UV-vis-NIR absorption and cyclic voltammetry data for P(OE3)-P(EH) and P(EH) undergoing repeated cycling. In contrast to P(OE3)-P(Me), the minimization of side-chain free volume in these two

polymers results in highly reversible electrochemical switching after the initial “break-in” period in the first few cycles.

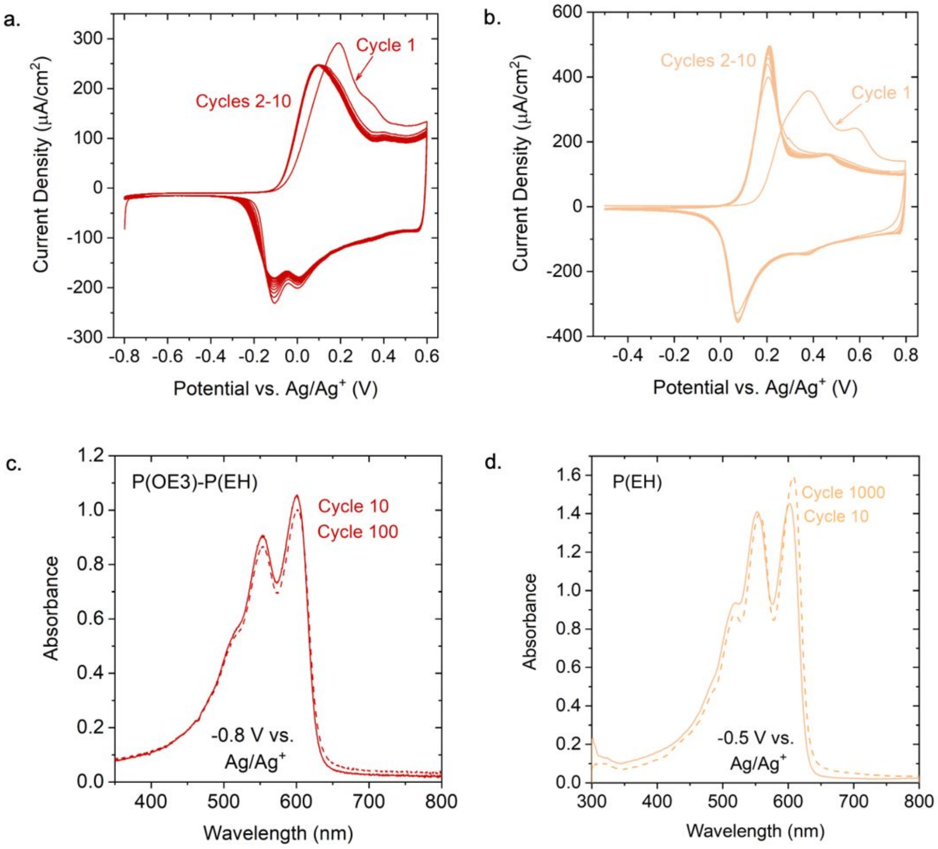

Figure S10. Cyclic voltammograms of **a.** P(OE3)-P(EH) and **b.** P(EH) during electrochemical cycling in 0.5 M tetrabutylammonium hexafluorophosphate ($TBA^+PF_6^-$) dissolved in degassed propylene carbonate (PC), showing near-constant redox currents upon cycling after the initial doping/dedoping sweeps. **c.** and **d.** Neutral-state UV-vis absorbance spectra for **c.** P(OE3)-P(EH) and **d.** P(EH) in the same electrolyte (0.5 M $TBA^+PF_6^-$/PC) revealing near-constant linear absorbance upon repeated cycling (cycle 10: solid lines, cycle 100 or 1000: dashed lines).

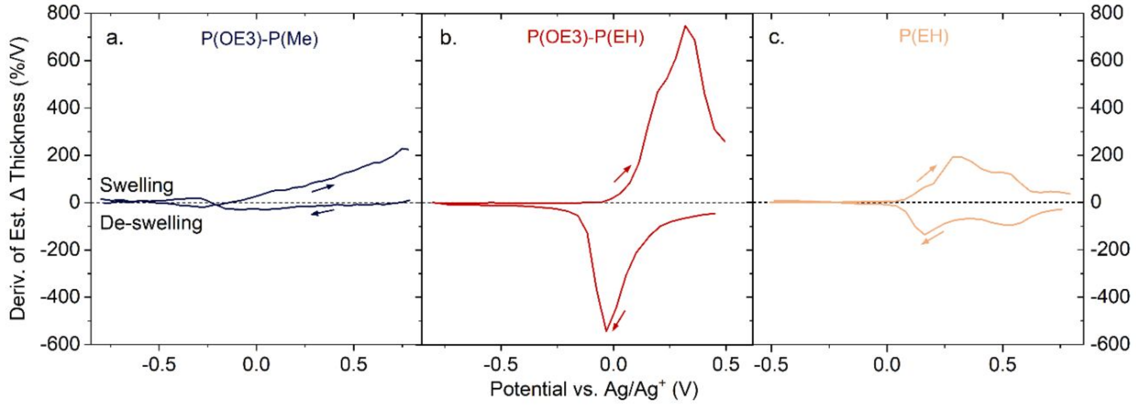

Figure S11. First derivative of thickness changes estimated from the 3rd overtone during the first electrochemical doping cycle for **a.** P(OE3)-P(Me), **b.** P(OE3)-P(EH), and **c.** P(EH), as measured via EQCM-D and shown in Figure 2g-i. Values above zero indicate positive thickness changes (swelling); values below zero (black dotted line) indicate negative thickness changes (de-swelling). Films cycled at 20 mV/second in 0.5 M PC/ $TBA^+PF_6^-$ electrolyte.

9. Commentary on small load approximation (Sauerbrey relation) for swelling analysis

After removing the bulk fluid shift, the measured QCM-D and EQCM-D frequency and dissipation shifts suggest that the Sauerbrey equation may be used to analyze passive and active swelling measurements. An increase in dissipation factor shifts, i.e., the extra damping of quartz crystal oscillations due viscous processes in the polymer film overlayer, are noted as expected for the more swollen polymer films. However, in our case these viscous losses are too small to cause significant deviations from the Sauerbrey mass-frequency shift relation for passive swelling, and the beginning of active swelling (1st doping-dedoping cycle). As detailed by Reviakine et al.[8], when $|\Delta \mathrm{D_n}/(\Delta \mathrm{f_n}/n)| \ll 4 \times 10^{-7}\mathrm{Hz}^{-1}$ for a 5 MHz crystal, the film is sufficiently rigid and any characteristic viscoelastic relaxations are sufficiently slow (compared to the high oscillation

frequencies range relevant to QCM measurement) to be approximated as a small, rigid load coupled to the quartz crystal, as done in the Sauerbrey approximation. The maximum value of $|\Delta D_3/(\Delta f_3/3)|$ is 4.7 $\times 10^{-8}\mathrm{Hz}^{-1}$ for P(OE3)-P(Me) at maximum active swelling (fifth doping cycle), while P(OE3)-P(EH) and P(EH) reach maximum $|\Delta D_3/(\Delta f_3/3)|$values of 1.2 $\times 10^{-8}\mathrm{Hz}^{-1}$ and 6.5 $\times 10^{-9}\mathrm{Hz}^{-1}$ respectively at maximum active swelling, well below the threshold of $4 \times 10^{-7}\mathrm{Hz}^{-1}$.

Equivalently, the Sauerbrey equation is accurate in cases where overtone number-normalized frequency shifts result in equal implied mass shifts. These conditions are met in both the passive and active swollen states in at least the first cycle for all polymers. Note that for the 96 nm P(OE3)-P(Me) film used in EQCM-D measurements, after the first cycle, the fundamental frequency and third overtone-implied thickness changes diverge as shown in Figure 3e, suggesting that the Sauerbrey relation is ***underestimating*** thickness in this highly swollen film after doping/dedoping, further emphasizing the role of electrolyte trapped in the films.

10. Doping/dedoping kinetics

The role of side chains on directing ion transport is further illustrated by examining the doping and dedoping kinetics of each polymer, measured via the absorbance of each film at the wavelength of maximum absorbance for the neutral π- π* absorption band in the UV-vis spectrum. Intriguingly, despite a notion that fast ion conductivity (as measured through doping/dedoping kinetics) is a direct consequence of the levels of electrolyte swelling in films[9,10] we see similar doping kinetics between these materials (Figures S12 and S13), despite the fact that the levels of passive swelling in these ProDOT materials differ by nearly two orders of magnitude.

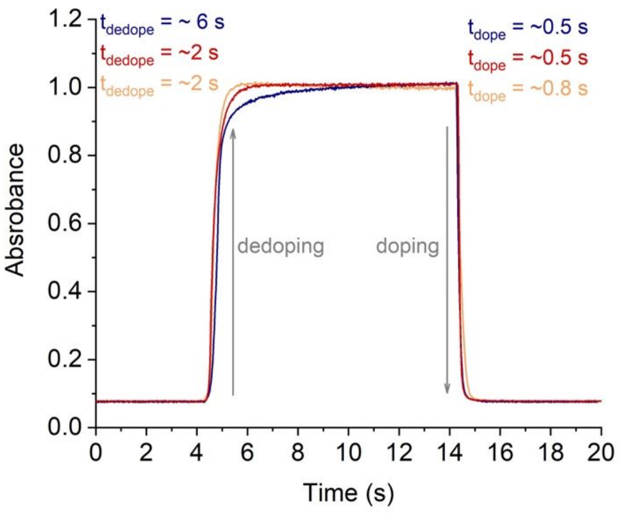

Figure S12. P(ProDOT) doping kinetics in organic electrolytes. Shown are P(OE3)-P(Me) (blue), P(OE3)-P(EH) (red), and P(EH) (peach) in 0.5 M $TBA^{+}PF_6^{-}$/PC. The absorption is measured at λ_{max} while the films are switched between the doped and dedoped states in 10 second intervals (-0.8 and 0.8 V for P(OE3)-P(Me) and P(EH), and -0.8 and 0.5 V for P(OE3)-P(EH)).

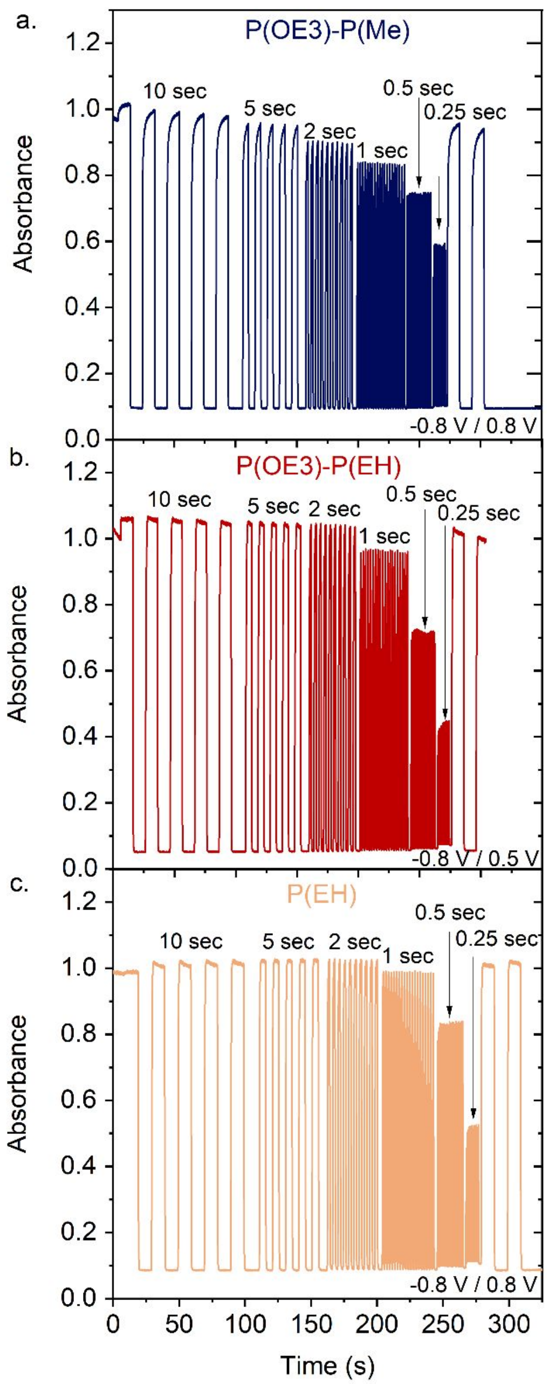

Figure S13. Optical doping/dedoping kinetics in 0.5M $TBA^+PF_6^-$/PC. **a.** P(OE3)-P(Me) switching between -0.8 and 0.8 V. **b.** P(OE3)-P(EH) switching between -0.8 and 0.5 V, **c.** P(EH) switching between -0.8 and 0.8 V. Potentials reported vs. Ag/Ag^+ (V).

11. Grazing Incidence Wide-Angle X-Ray Scattering (GIWAXS)

Grazing-incidence wide-angle X-ray scattering (GIWAXS) data for P(EH) is shown in Figure S14 as an example for the three polymers investigated here. The GIWAXS q-image for P(EH) features broad (100) and (010) diffraction features, indicating pronounced disorder, i.e., significant lack of long-range order. The GIWAXS spectrum for P(EH) was collected at the 11-BM Complex Materials Scattering beamline at Brookhaven National Laboratory's National Synchrotron Light Source II, managed by beamline scientist Dr. Ruipeng Li. The pattern was captured from a high fluence 13.5 keV X-ray beam delivered from a 3-pole wiggler source at an angle of incidence of approximately 0.14°. P(EH) was spin-coated on a polished Si wafer, and then placed onto a multi-sample holder bar and adhered via silicone vacuum grease. The sample was then mounted into a robotic sample exchanger in a vacuum-sealed housing and aligned with the X-ray beam. Raw WAXS data was taken from a dedicated in-vacuum detector (DECTRIS Pilatus 800K) with a CCD pixel size of 0.172 mm. A standard sample of silver behenate (AgBH) was also measured to determine the beam center and sample-detector distance. Raw WAXS data was analyzed using the Nika 2D SAS Macros available in Igor Pro 9 for conversion into the q-image shown below. [12] Similar results are reported for P(OE3)-P(Me) and P(OE3)-P(EH), spin-coated on polished Si wafers (see Ref.11; accessible at http://hdl.handle.net/1853/66407).

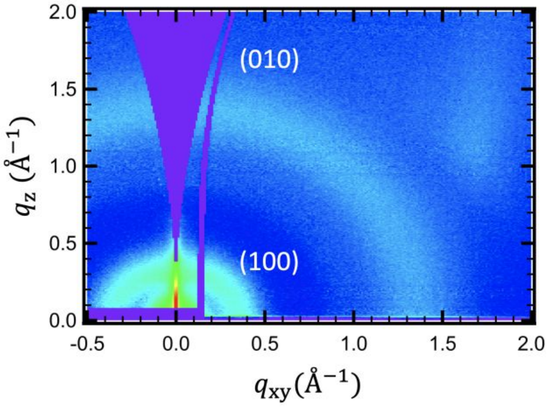

Figure S14. Grazing incidence wide-angle X-ray scattering *q*-image for P(EH). 0.14° angle of incidence. Sample characterized as-cast from films spin coated on Silicon wafer substrates.

12. Fast Scanning Calorimetry

Fast Scanning Calorimetry was conducted under nitrogen using a Mettler Toledo Flash DSC 1 equipped with a Huber TC100 cooler to control the temperature between -90 and +450 °C. Powder samples were deposited onto Mettler Toledo Standard MultiSTAR UFS 1 chips and measured at rates of 4000 K/second, using the temperature program described previously in detail in Refs.13–15 and shown in Figure S15a (right panel).

Figure S15a provides example F-DSC measurements on the ubiquitous and widely studied poly(3-hexyl thiophene), P3HT. Plotted are data for a sample that first was measured unaged (red) and, then aged (blue). Since melting is a first-order transition, no difference in the thermograms are observed, whether aged or unaged. The melting transition is indicated in panel 1 with a blue arrow labelled "T_m". In contrast, other transitions lead to an enthalpic overshoot in the thermogram after aging, but not in the heating scan where no aging was used. In materials such as P3HT this allows for the detection of relevant temperatures such as the glass transition temperature[16] (indicated in Figure S15a with a red arrow), or features that are attributed to side-chain softening when materials with more complex side-chain motifs are investigated.[13,14]

For the three P(ProDOT)s studied here, no strong endotherms nor enthalpic overshoots were observed, indicating high molecular disorder both of the backbone and the side chains. This is beneficial for this study as it allowed us to study structure/property interrelationships without the complication of different degrees of structural order in the materials.

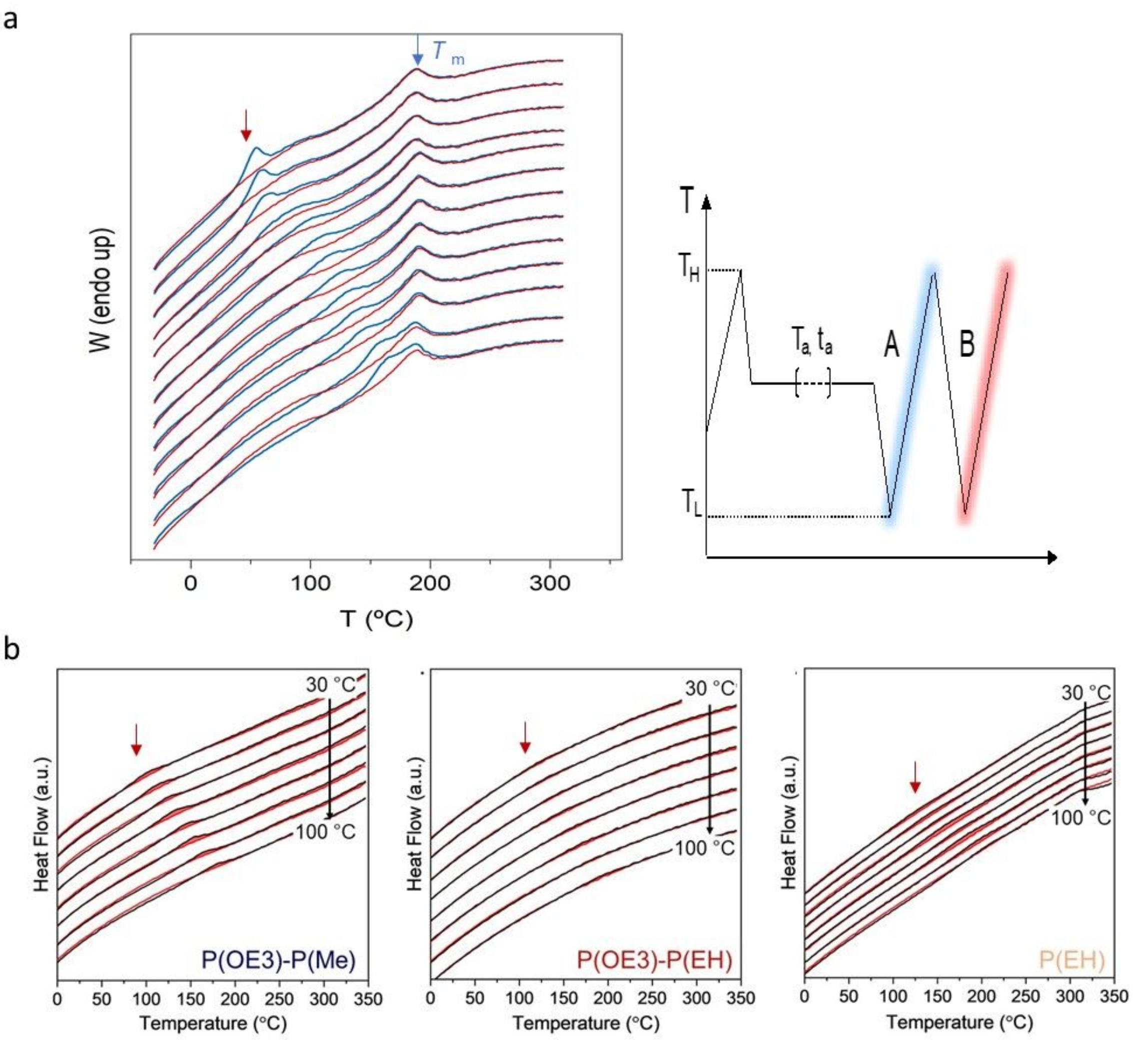

Figure S15. a. Fast calorimetry (F-DSC) data for the well-studied polymer semiconductor P3HT (left) and temperature program used to measure even very small phase transitions not detectable in more standard methodologies (see for details Refs. 12-14). **b.** F-DSC thermograms for samples that were first measured without aging (red) and then after aging (black) show only very minute, hardly discernable enthalpic overshoots, indicating the presence of a weak transition with minute structural changes, such as that observed in liquid crystalline materials.

References

(1) Savagian, L. R.; Österholm, A. M.; Ponder, J. F.; Barth, K. J.; Rivnay, J.; Reynolds, J. R. Balancing Charge Storage and Mobility in an Oligo(Ether) Functionalized Dioxythiophene Copolymer for Organic- and Aqueous- Based Electrochemical Devices and Transistors. *Adv. Mater.* **2018**, *30* (50), 1804647. https://doi.org/10.1002/adma.201804647.

(2) Estrada, L. A.; Deininger, J. J.; Kamenov, G. D.; Reynolds, J. R. Direct (Hetero)Arylation Polymerization: An Effective Route to 3,4-Propylenedioxythiophene-Based Polymers with Low Residual Metal Content. *ACS Macro Lett.* **2013**, *2* (10), 869–873. https://doi.org/10.1021/mz4003886.

(3) Kerszulis, J. A.; Johnson, K. E.; Kuepfert, M.; Khoshabo, D.; Dyer, A. L.; Reynolds, J. R. Tuning the Painter's Palette: Subtle Steric Effects on Spectra and Colour in Conjugated Electrochromic Polymers. *J. Mater. Chem. C* **2015**, *3* (13), 3211–3218. https://doi.org/10.1039/C4TC02685C.

(4) Remmele, J.; Shen, D. E.; Mustonen, T.; Fruehauf, N. High Performance and Long-Term Stability in Ambiently Fabricated Segmented Solid-State Polymer Electrochromic Displays. *ACS Appl. Mater. Interfaces* **2015**, *7* (22), 12001–12008. https://doi.org/10.1021/acsami.5b02090.

(5) Easley, A. D.; Ma, T.; Eneh, C. I.; Yun, J.; Thakur, R. M.; Lutkenhaus, J. L. A Practical Guide to Quartz Crystal Microbalance with Dissipation Monitoring of Thin Polymer Films. *J. Polym. Sci.* **2022**, *60* (7), 1090–1107. https://doi.org/10.1002/pol.20210324.

(6) Hollingsworth, N. R.; Wilkanowicz, S. I.; Larson, R. G. Salt- And PH-Induced Swelling of a Poly(Acrylic Acid) Brush: Via Quartz Crystal Microbalance w/Dissipation (QCM-D). *Soft Matter* **2019**, *15* (39), 7838–7851. https://doi.org/10.1039/c9sm01289c.

(7) Schmode, P.; Savva, A.; Kahl, R.; Ohayon, D.; Meichsner, F.; Dolynchuk, O.; Thurn-Albrecht, T.; Inal, S.; Thelakkat, M. The Key Role of Side Chain Linkage in Structure Formation and Mixed Conduction of Ethylene Glycol Substituted Polythiophenes. *ACS Appl. Mater. Interfaces* **2020**, *12* (11), 13029–13039. https://doi.org/10.1021/acsami.9b21604.

(8) Reviakine, I.; Johannsmann, D.; Richter, R. P. Hearing What You Cannot See and Visualizing What You Hear: Interpreting Quartz Crystal Microbalance Data from Solvated Interfaces. *Anal. Chem.* **2011**, *83* (23), 8838–8848. https://doi.org/10.1021/ac201778h.

(9) Wieland, M.; Dingler, C.; Merkle, R.; Maier, J.; Ludwigs, S. Humidity-Controlled Water Uptake and Conductivities in Ion and Electron Mixed Conducting Polythiophene Films.

ACS Appl. Mater. Interfaces **2020**, *12* (5), 6742–6751. https://doi.org/10.1021/acsami.9b21181.

(10) Savva, A.; Hallani, R.; Cendra, C.; Surgailis, J.; Hidalgo, T. C.; Wustoni, S.; Sheelamanthula, R.; Chen, X.; Kirkus, M.; Giovannitti, A.; Salleo, A.; McCulloch, I.; Inal, S. Balancing Ionic and Electronic Conduction for High-Performance Organic Electrochemical Transistors. *Adv. Funct. Mater.* **2020**, *30* (11), 1907657. https://doi.org/10.1002/adfm.201907657.

(11) Savagian, L. R. Establishing Design Rules for Polythiophenes Used in Electrochemical Applications, Georgia Institute of Technology, 2021.

(12) Ilavsky, J. Nika : Software for Two-Dimensional Data Reduction. *J. Appl. Crystallogr.* **2012**, *45* (2), 324–328. https://doi.org/10.1107/S0021889812004037.

(13) Sommerville, P. J. W.; Balzer, A. H.; Lecroy, G.; Guio, L.; Wang, Y.; Onorato, J. W.; Kukhta, N. A.; Gu, X.; Salleo, A.; Stingelin, N.; Luscombe, C. K. Influence of Side Chain Interdigitation on Strain and Charge Mobility of Planar Indacenodithiophene Copolymers. *ACS Polym. Au* **2023**, *3* (1), 59–69. https://doi.org/10.1021/acspolymersau.2c00034.

(14) Sommerville, P. J. W.; Li, Y.; Dong, B. X.; Zhang, Y.; Onorato, J. W.; Tatum, W. K.; Balzer, A. H.; Stingelin, N.; Patel, S. N.; Nealey, P. F.; Luscombe, C. K. Elucidating the Influence of Side-Chain Circular Distribution on the Crack Onset Strain and Hole Mobility of Near-Amorphous Indacenodithiophene Copolymers. *Macromolecules* **2020**, *53* (17), 7511–7518. https://doi.org/10.1021/acs.macromol.0c00512.

(15) Matrone, G. M.; Gutiérrez-Meza, E.; Balzer, A. H.; Khirbat, A.; Levitsky, A.; Sieval, A. B.; Frey, G. L.; Richter, L. J.; Silva, C.; Stingelin, N. The Hole in the Bucky: Structure–Property Mapping of Closed- vs. Open-Cage Fullerene Solar-Cell Blends via Temperature/Composition Phase Diagrams. *J. Mater. Chem. C* **2021**, *9* (45), 16304–16312. https://doi.org/10.1039/D1TC03082E.

(16) Martín, J.; Stingelin, N.; Cangialosi, D. Direct Calorimetric Observation of the Rigid Amorphous Fraction in a Semiconducting Polymer. *J. Phys. Chem. Lett.* **2018**, *9* (5), 990–995. https://doi.org/10.1021/acs.jpclett.7b03110.
